# Safety and High Level Efficacy of the Combination Malaria Vaccine Regimen of RTS,S/AS01_B_ With Chimpanzee Adenovirus 63 and Modified Vaccinia Ankara Vectored Vaccines Expressing ME-TRAP

**DOI:** 10.1093/infdis/jiw244

**Published:** 2016-06-15

**Authors:** Tommy Rampling, Katie J. Ewer, Georgina Bowyer, Carly M. Bliss, Nick J. Edwards, Danny Wright, Ruth O. Payne, Navin Venkatraman, Eoghan de Barra, Claudia M. Snudden, Ian D. Poulton, Hans de Graaf, Priya Sukhtankar, Rachel Roberts, Karen Ivinson, Rich Weltzin, Bebi-Yassin Rajkumar, Ulrike Wille-Reece, Cynthia K. Lee, Christian F. Ockenhouse, Robert E. Sinden, Stephen Gerry, Alison M. Lawrie, Johan Vekemans, Danielle Morelle, Marc Lievens, Ripley W. Ballou, Graham S. Cooke, Saul N. Faust, Sarah Gilbert, Adrian V. S. Hill

**Affiliations:** 1 The Jenner Institute; 2 Centre for Statistics in Medicine, University of Oxford; 3 Department of Life Sciences; 4 Infectious Diseases Section, Faculty of Medicine, Department of Medicine, Imperial College London; 5 NIHR Wellcome Trust Clinical Research Facility, University of Southampton and University Hospital Southampton NHS Foundation Trust, United Kingdom; 6 Royal College of Surgeons in Ireland, Dublin; 7 PATH Malaria Vaccine Initiative, Seattle, Washington; 8 GSK Vaccines, Rixensart, Belgium

**Keywords:** malaria, *P. falciparum*, vaccine, RTS,S, ChAd63, ME-TRAP

## Abstract

***Background.*** The need for a highly efficacious vaccine against *Plasmodium falciparum* remains pressing. In this controlled human malaria infection (CHMI) study, we assessed the safety, efficacy and immunogenicity of a schedule combining 2 distinct vaccine types in a staggered immunization regimen: one inducing high-titer antibodies to circumsporozoite protein (RTS,S/AS01_B_) and the other inducing potent T-cell responses to thrombospondin-related adhesion protein (TRAP) by using a viral vector.

***Method.*** Thirty-seven healthy malaria-naive adults were vaccinated with either a chimpanzee adenovirus 63 and modified vaccinia virus Ankara–vectored vaccine expressing a multiepitope string fused to TRAP and 3 doses of RTS,S/AS01_B_ (group 1; n = 20) or 3 doses of RTS,S/AS01_B_ alone (group 2; n = 17). CHMI was delivered by mosquito bites to 33 vaccinated subjects at week 12 after the first vaccination and to 6 unvaccinated controls.

***Results.*** No suspected unexpected serious adverse reactions or severe adverse events related to vaccination were reported. Protective vaccine efficacy was observed in 14 of 17 subjects (82.4%) in group 1 and 12 of 16 subjects (75%) in group 2. All control subjects received a diagnosis of blood-stage malaria parasite infection. Both vaccination regimens were immunogenic. Fourteen protected subjects underwent repeat CHMI 6 months after initial CHMI; 7 of 8 (87.5%) in group 1 and 5 of 6 (83.3%) in group 2 remained protected.

***Conclusions.*** The high level of sterile efficacy observed in this trial is encouraging for further evaluation of combination approaches using these vaccine types.

**Clinical Trials Registration.** NCT01883609.

Malaria remains one of the leading causes of mortality globally [1], and there is urgent need for a vaccine. The majority of deaths are in children <5 years old, with this age group accounting for approximately 306 000 deaths in 2015. The enormous economic and social consequences of malaria have been well documented [2]. Efforts to develop effective vaccines are complicated by the complex immunology of malaria parasite infection, and no reliable natural model of complete immunity exists. Despite this, a small number of candidate vaccines have shown partial efficacy against experimental and natural human infection, with the current leading vaccine being the recombinant protein in adjuvant, RTS,S/AS01. RTS,S targets circumsporozoite protein (CS), which is expressed by the *Plasmodium falciparum* sporozoite at the preerythrocytic stage and was the first subunit vaccine to show high rates of sterile efficacy, typically 50%, in controlled human malaria infection (CHMI) studies [3]. In a large African phase 3 trial, this vaccine had an efficacy against clinical malaria of 55.8% (97.5% confidence interval [CI], 50.6%–60.4%) in children aged 5–17 months and 31.3% (23.6%–38.3%) in infants aged 6–12 weeks over the first year after vaccination [4, 5]. Vaccine efficacy wanes over time but can be enhanced by a fourth dose [6]. Analysis of the immunological correlates of efficacy of this vaccine suggest that vaccine-induced antibodies targeting CS are the most important mediators of RTS,S-induced protection against malaria [3], although no antibody level threshold has been shown to be predictive of efficacy. The rate at which anti-CS antibodies wane is similar to the rate at which efficacy declines [7, 8], suggesting that anti-CS antibodies may also be associated with the duration of protection. A number of factors, including age at vaccination, human immunodeficiency virus status, and high baseline anti-CS antibody titers influence anti-CS antibody titers after vaccination with RTS,S [9].

The preerythrocytic stage of *P. falciparum* infection presents an attractive target for an efficacious human vaccine because sufficient reduction in the number of viable merozoites reaching the blood from the liver will prevent parasitization of red blood cells and initiation of the symptomatic blood stage of infection. Anti-CS antibodies can target sporozoites for destruction prior to hepatocyte invasion. Because sporozoites travel from the skin to liver within minutes, it may be difficult for a vaccine to achieve complete protection against *P. falciparum* based solely on antibodies to sporozoites. The liver stage of infection provides a longer window of opportunity for cell-mediated immunity to recognize and destroy infected hepatocytes. Chimpanzee adenovirus 63 (ChAd63) with a multiepitope string fused to thrombospondin-related adhesion protein (ME-TRAP) insert and modified vaccinia virus Ankara (MVA) with the ME-TRAP insert are viral-vectored vaccines, and when they are administered in a prime-boost sequence at an 8-week interval, they are a leading candidate vaccine strategy targeting the liver stage of infection [10]. The ChAd63 and MVA viral vectors deliver the recombinant ME-TRAP insert, which generates a potent cellular immune response against the liver-stage *P. falciparum* antigen, TRAP, of greater magnitude than the cellular response induced by RTS,S/AS01. This strategy showed durable partial efficacy in 2 phase 2a sporozoite challenge trials in the United Kingdom [11, 12], using the 3D7 parasite as a challenge strain. The viral vector–encoded *P. falciparum* TRAP allele is from the heterologous T9/96 strain, and induced T-cell responses correlate with efficacy [11]. Therefore, these are effectively heterologous strain CHMI studies. Interestingly, a higher level of efficacy of 67% (95% CI, 33%–83%) against *P. falciparum* infections detected by polymerase chain reaction (PCR) was observed in a phase 2b trial in Kenyan adults [13]. Again, T cells to TRAP peptides correlated with vaccine efficacy, but the short duration of malaria transmission and follow-up at this trial site precluded analysis of the durability of vaccine-induced protection [13]. This heterologous prime-boost strategy showed potent cellular immunogenicity in adults in the United Kingdom [11], as well as adults and infants in malaria-endemic areas [13–15] (Ewer et al, unpublished data) and has an excellent track record of safety and tolerability in these populations. Analysis of the potential utility of combining antisporozoite and anti–liver-stage vaccines have suggested a likely additive or synergistic effect [16], in keeping with findings from preclinical studies [17, 18].

In this phase 1/2a, open-labeled, CHMI study, we assessed the safety, immunogenicity, and efficacy of a vaccine schedule combining these 2 distinct candidate vaccine types in a staggered immunization regimen: one that induces very high titer antibodies to CS, using RTS,S/AS01_B_, and another that induces potent T-cell responses to TRAP, using viral vectors.

## METHODS

### Participants

Recruitment and vaccination was conducted at 3 United Kingdom sites, in Oxford, Southampton and London. The CHMI procedure was performed as previously described [19] at Imperial College, London, using 5 infectious bites from *Anopheles stephensi* mosquitoes infected with *P. falciparum* strain 3D7. All subjects were infected with a single batch of mosquitoes at the initial CHMI and with a second single batch at the repeat CHMI. Infected mosquitoes were supplied by the Department of Entomology, Walter Reed Army Institute of Research (Washington D.C.). Healthy, malaria-naive males and nonpregnant females aged 18–45 years were invited to participate in the study. All volunteers gave written informed consent prior to participation, and the study was conducted according to the principles of the Declaration of Helsinki and in accordance with good clinical practice (GCP).

### Ethical and Regulatory Approval

Necessary approvals for the study were granted by the United Kingdom National Research Ethics Service, Committee South Central–Oxford A (reference 13/SC/0208), the Western Institution Review Board (reference 20130698), and the United Kingdom Medicines and Healthcare Products Regulatory Agency (reference 21584/0317/001-0001). The trial was registered with ClinicalTrials.gov (reference NCT01883609). The Local Safety Committee provided safety oversight, and GCP compliance was independently monitored externally by the Clinical Trials and Research Governance Team of the University of Oxford.

### Study Design

This phase 2a, open-labeled, partially randomized challenge trial consisted of 4 cohorts. Allocation to study group occurred at screening and was based on subject preference. Any subjects without a preference were randomly assigned to vaccine group 1 or vaccine group 2. Group 1 (n = 20) received 5 vaccinations (RTS,S/AS01_B_ 50 µg at 0, 4, and 8 weeks, ChAd63 ME-TRAP 5 × 10^10^ virus particles at 2 weeks, and MVA ME-TRAP 2 × 10^8^ plaque-forming units at 10 weeks); group 2 (n = 20) received 3 vaccinations (RTS,S/AS01_B_ 50 µg at 0, 4, and 8 weeks); and group 3 (n = 6) received no vaccinations. All vaccinations were administered intramuscularly into the deltoid region of the arm. In each volunteer, all RTS,S/AS01_B_ injections were given in one arm, and all viral vector injections were given in the contralateral arm. All subjects underwent initial CHMI by mosquito bite at the same time (week 12 after first vaccination for vaccinated subjects). Following CHMI, a diagnosis of blood-stage malaria parasite infection was made in subjects with symptoms suggestive of malaria and positive findings of thick film microscopy or, if either thick film was negative or symptoms were absent, in subjects with a qPCR result of >500 parasites/mL [12]. Vaccinated subjects who had not developed blood-stage malaria by day 21 after CHMI were deemed to exhibit sterile protection and were invited to undergo repeat CHMI 6 months later, for which an additional control group (group 4) was recruited.

Further details of the study sites, inclusion/exclusion criteria, vaccines, clinical follow-up, safety monitoring, malaria treatment and diagnosis, immunological and molecular methods, and statistical analysis can be found in the Supplementary Materials.

## RESULTS

### Participants

**Figure 1.**
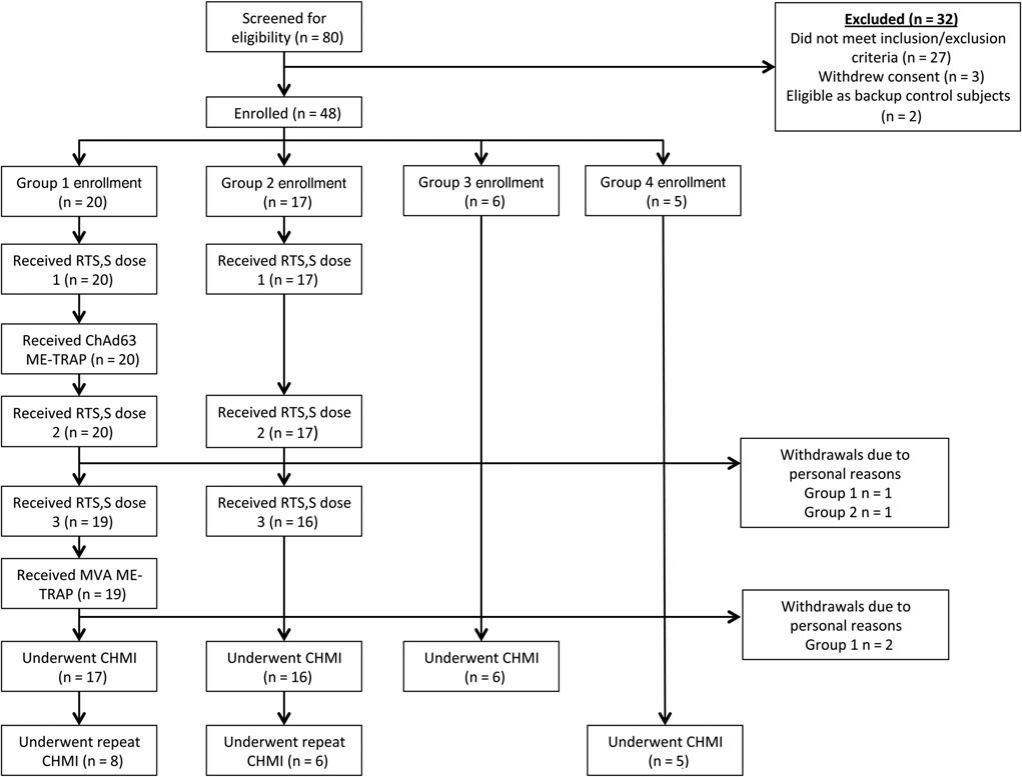
Flow of study design and volunteer recruitment. Twenty-seven subjects were excluded because of inclusion/exclusion criteria. Three subjects withdrew consent after screening but before enrollment. Two subjects were deemed eligible as control subjects but only after group 3 enrollment was complete. They were kept as backup subjects in case of last-minute withdrawals from group 3 but never underwent controlled human malaria infection (CHMI). Seventeen subjects expressed a preference as to which vaccine group to be allocated to and were assigned accordingly. Twenty subjects expressed no preference for vaccine group allocation, and were therefore randomized to group by the study statistician. Abbreviations: ChAd63, chimpanzee adenovirus serotype 63; ME-TRAP, multiple-epitope thrombospondin-related adhesion protein; MVA, modified vaccinia virus Ankara.

Eighty subjects were screened for eligibility, and 48 subjects were identified as eligible. Twenty subjects were allocated to group 1 to receive RTS,S/AS01_B_ and viral vectors encoding ME-TRAP. Seventeen subjects were allocated to group 2 to receive RTS,S/AS01_B_ only. Six unvaccinated controls were recruited to group 3 for the initial CHMI, and 5 subjects were allocated to group 4 for the repeat CHMI. Vaccinations took place between 2 September 2013 and 13 November 2013. Prior to CHMI, 3 subjects withdrew from group 1, and 1 subject withdrew from group 2. There were no withdrawals due to safety concerns, and no predefined study stopping or holding rules were activated. CHMI was performed on 25 and 26 November 2013, and repeat CHMI was performed on 13 May 2014 (Figure 1).

### Protective Efficacy Against CHMI

**Figure 2.**
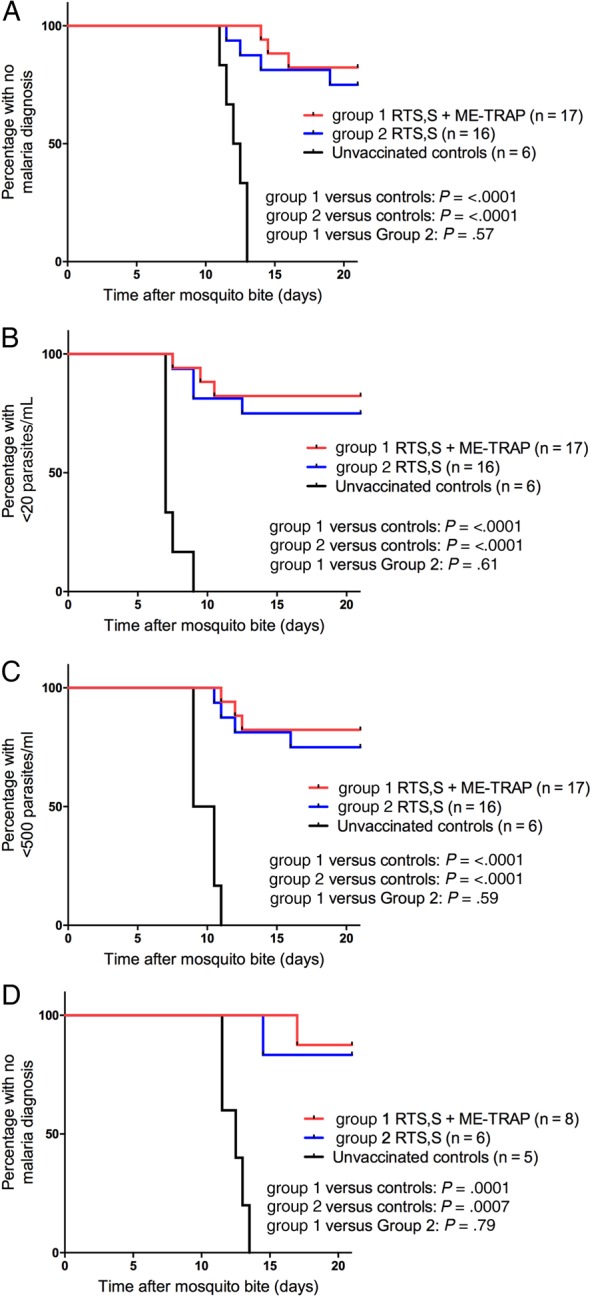
Efficacy of RTS,S/AS01_B_ plus chimpanzee adenovirus 63 and modified vaccinia virus Ankara–vectored vaccine (ChAd63-MVA) expressing a multiepitope string fused to thrombospondin-related adhesion protein (ME-TRAP) and RTS,S/AS01_B_ alone following *Plasmodium falciparum* 3D7 sporozoite challenge. *A*, Kaplan–Meier survival analysis of the time to treatment following initial controlled human malaria infection (CHMI). Mean time to diagnosis (± standard deviation [SD]) was 12.2±0.7 days for unvaccinated controls. Seventeen of 17 subjects (100%) in group 1 and 14 of 16 subjects (87.5%) in group 2 had no diagnosis by day 21 or received a diagnosis after the control mean time + 2 SD. *B*, Kaplan–Meier survival analysis of the time to the first sample with >20 parasites/mL detected by quantitative polymerase chain reaction (qPCR). Mean time to end point (±SD) was 7.4±0.7 days for unvaccinated controls. Sixteen of 17 subjects (94.1%) in group 1 and 15 of 16 subjects (93.8%) in group 2 did not reach this end point or did so after the control mean time + 2 SD. *C*, Kaplan–Meier survival analysis of the time to the first sample with >500 parasites/mL detected by qPCR. Mean time to end point (±SD) was 9.8±0.8 days for unvaccinated controls. Seventeen of 17 subjects (100%) in group 1 and 15 of 16 subjects (93.8%) in group 2 did not reach this end point or did so after the control mean time + 2 SD. *D*, Kaplan–Meier survival analysis of the time to treatment following repeat CHMI in protected subjects. Significance testing was performed by the log-rank test.

A total of 39 subjects participated in the initial CHMI (17 subjects from group 1, 16 subjects from group 2, and 6 subjects from group 3), which was conducted over 2 days. Three subjects in group 1 and 4 subjects in group 2 received a diagnosis of malaria before day 21 after challenge, resulting in a sterile efficacy of 82.4% (95% CI, 64%–100%) and 75% (95% CI, 54–96), respectively (Figure 2). The median time to diagnosis was 14.5 days in group 1 and 13.25 days in group 2. All 6 control subjects received a diagnosis of malaria, with a median time to diagnosis of 12.25 days (range, 11–13 days; SD, 0.7 days). Both vaccine regimens demonstrated a significantly reduced risk of malaria parasite infection over controls in the per protocol analysis (group 1 hazard ratio [HR], 0.065; *P* < .0001; group 2 HR, 0.12; *P* < .0001 for group 2), but there was no significant difference in efficacy between vaccine regimen (HR, 0.65; *P* .57). Eight protected subjects from group 1 and 6 protected subjects from group 2 agreed to undergo repeat CHMI. A single subject each from group 1 and group 2 received a diagnosis of malaria, on day 17 and day 14.5, respectively, and all 5 control subjects developed malaria, with a mean time to diagnosis of 12.4 days (median, 12.5 days; range, 11.5–13.5 days; SD, 0.8 days).

### Safety

The safety profile of a 3-dose regimen of RTS,S/AS01_B_ and of ChAd63-MVA ME-TRAP when given separately to malaria-naive adults has been described previously [3, 10–12, 20], and a similar reactogenicity profile was observed after vaccination in this study. The majority of adverse events (AEs) following vaccinations in both group 1 and group 2 were mild in severity and self-limiting. There were no serious AEs related to vaccination, and no suspected, unexpected serious adverse reactions (SUSARs). Solicited and unsolicited AEs following vaccination are detailed in Supplementary Tables 1–12.

### Humoral Response to Vaccination

**Figure 3.**
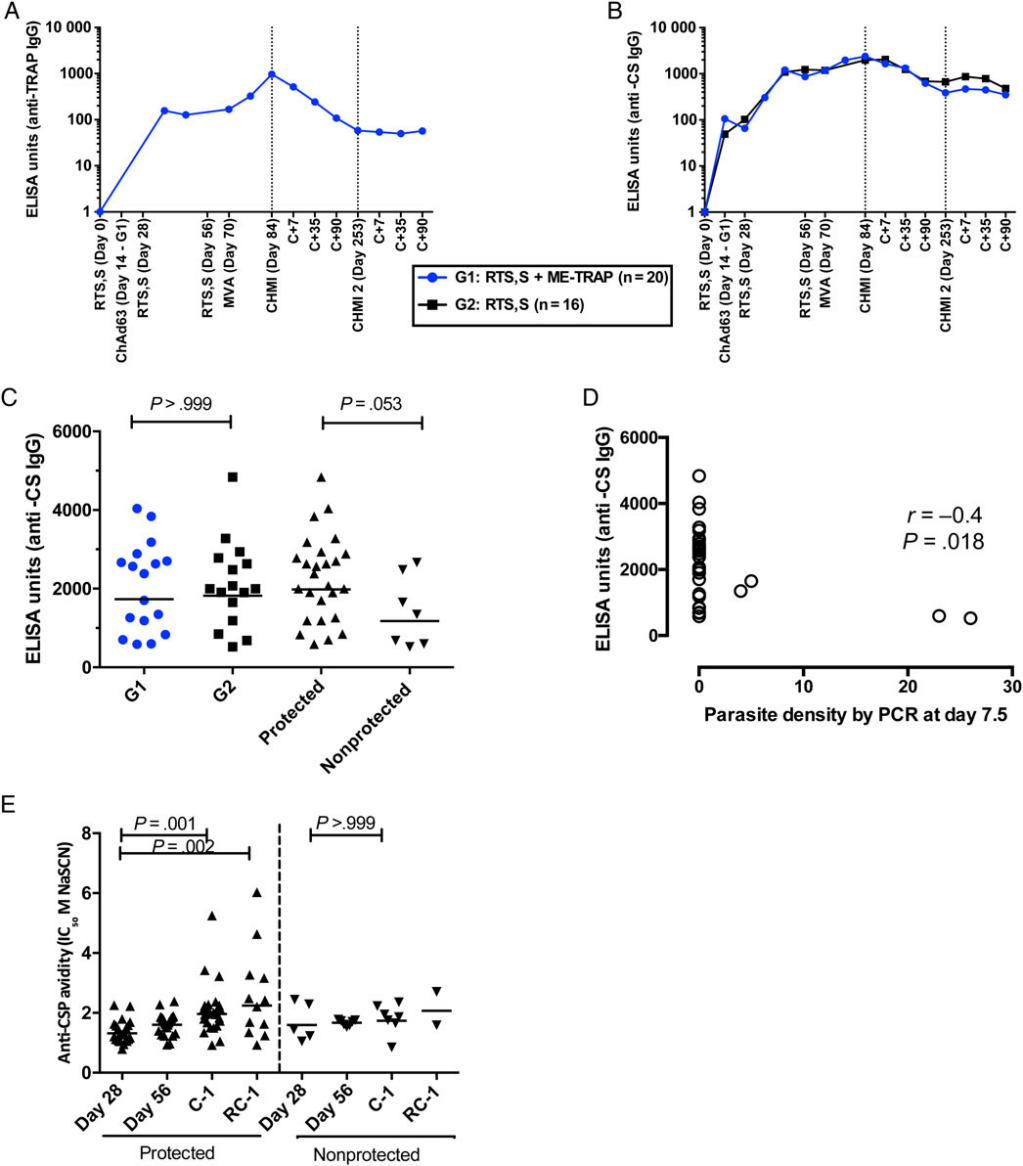
Antibody responses to vaccination, measured by enzyme-linked immunosorbent assay (ELISA). *A*, Anti-thrombospondin adhesion protein (TRAP) immunoglobulin G (IgG) antibody responses after vaccination with RTS,S/AS01_B_ plus chimpanzee adenovirus 63 and modified vaccinia virus Ankara–vectored vaccine (ChAd63-MVA) expressing a multiepitope string fused to TRAP (ME-TRAP; group 1 subjects only). Lines represent group medians. *B*, Anti–circumsporozoite protein (CS) IgG antibody responses after vaccination with RTS,S/AS01_B_ plus ChAd63-MVA ME-TRAP (group 1; blue) or RTS,S alone (group 2; black). Line represents group median. *C*, Comparison of anti-CS IgG antibody responses between group 1 (blue) and group 2 (black) as measured on the day before controlled human malaria infection (CHMI). *P* > .999, by the Mann–Whitney test. Comparison of anti-CS IgG antibody responses in volunteers who were or were not sterilely protected. Lines represent geometric means. *D*, Correlation between anti-CS IgG titers on the day before challenge and parasite density on day 7 after challenge. Spearman *r* = −0.4; *P* = .018. *E*, Avidity of total IgG against the NANP repeat region of circumsporozoite protein. Significant increase in avidity between day 28 and the day before challenge in protected but not nonprotected volunteers. *P* = .001, by the Wilcoxon matched pairs test. Avidity of total IgG remained significantly higher at time of the second CHMI (RC-1) than at day 28. *P* = .002, Mann–Whitney test. Lines represent geometric mean. Abbreviations: C+, elapsed time after CHMI, in days; C-1, day before CHMI; PCR, polymerase chain reaction; RC-1, day before second CHMI.

Anti-TRAP IgG antibodies were measured in group 1 subjects only (Figure 3), and geometric mean titers (GMTs) peaked on the day before challenge, at 947 ELISA units (EU; 95% CI, 617–1455). No association was detected between anti-TRAP IgG levels and efficacy (Spearman *r* = −0.25; *P* = .3). Anti-CS antibodies were measured at key time points in all vaccinated subjects. Serum anti-CS antibody levels peaked on the day before challenge in both vaccinated groups, with peak GMTs of 1733 EU (95% CI, 1240–2422) and 1824 EU (95% CI, 1330–2502) in groups 1 and 2, respectively. There was no significant difference in anti-CS antibody GMTs between group 1 and group 2 on the day before challenge (*P* > .99, by the Mann–Whitney test). Anti-CS antibody GMTs on the day before challenge were significantly higher in protected subjects (1985 EU [95% CI, 1584–2487]), compared with those in nonprotected subjects (1177 [95% CI, 627–2209]; *P* = .035, by the Mann–Whitney test; Figure 3). There was a correlation between anti-CS antibody titer and parasite density on day 7.5 (Spearman *r* = −0.4; *P* = .018). There was no significant difference in the avidity of anti-CS antibodies between protected and nonprotected volunteers at any time point, but avidity significantly increased between day 28 and the day before challenge in protected but not nonprotected volunteers (*P* = .001 and *P* > .99, respectively, by the Wilcoxon matched pairs test). Avidity also increased between day 56 and the day before challenge in protected but not nonprotected volunteers (*P* < .0001 and *P* = .375, respectively, by the Wilcoxon matched pairs test). In the protected vaccinated subjects who underwent repeat CHMI, avidity on the day before rechallenge remained significantly higher than at day 28 (*P* = .002, by the Mann–Whitney test).

### Cellular Response to Vaccination

**Figure 4.**
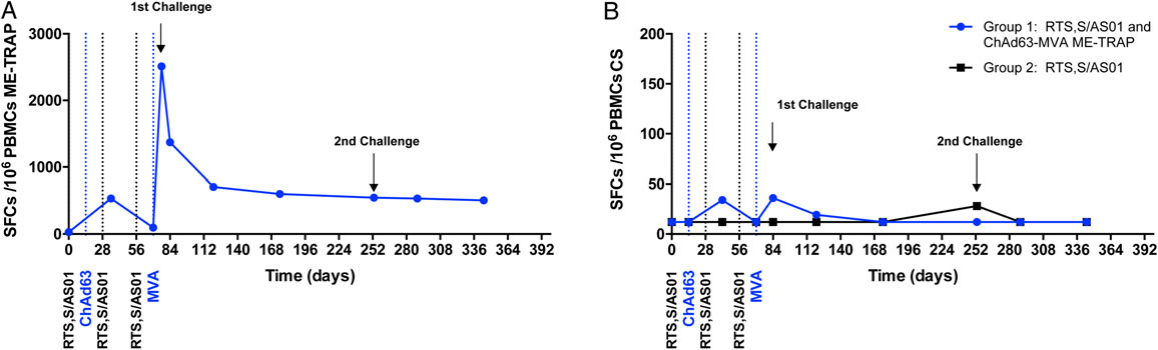
Antigen-specific T-cell responses to vaccination, measured by interferon γ (IFN-γ) enzyme-linked immunospot assay (ELISPOT). *A*, Median T-cell responses to multiepitope string fused to thrombospondin-related adhesion protein (ME-TRAP). *B*, Median T-cell responses to circumsporozoite protein (CS) peptide pools are shown for group 1 (RTS,S/AS01 and ME-TRAP; blue line) and group 2 (RTS,S/AS01; black line). Abbreviations: ChAd63, chimpanzee adenovirus 63; MVA, modified vaccinia virus Ankara; PBMC, peripheral blood mononuclear cell; SFC, spot-forming cell.

T-cell responses against ME-TRAP were measured in all group 1 subjects by an ex vivo interferon γ (IFN-γ) enzyme-linked immunosorbent spot (ELISPOT) assay (Figure 4). Peak responses after ChAd63 ME-TRAP vaccination were detected 21 days later (Geometric Mean, 539 spot-forming cells [SFCs] per million peripheral blood mononuclear cells [PBMCs]; 95% CI, 300–968 SFCs per million PBMCs). Peak responses after MVA ME-TRAP vaccination were detected 7 days later (median, 1520 SFCs per million PBMCs; interquartile range [IQR], 699–3305 SFCs per million PBMCs). T-cell responses against ME-TRAP were well maintained over time, with a median of 464 SFCs per million PBMCs (IQR, 231–933 SFCs per million PBMCs) 90 days after initial challenge and 342 SFCs per million PBMCs (IQR, 143–815 SFCs per million PBMCs) in participating subjects the day before repeat CHMI.

T-cell responses against CS were measured in all vaccinated subjects by an IFN-γ ELISPOT assay (Figure 4). Responses peaked in group 1 on the day before challenge (4 weeks after final dose of RTS,S/AS01_B_; median, 36 SFCs per million PBMCs; IQR, 12–176 SFCs per million PBMCs), with a median response of 12 SFCs per million PBMCs (IQR, 12–70 SFCs per million PBMCs) in group 2 at the same time point. No association between IFN-γ ELISPOT responses to TRAP or CS and vaccine efficacy was detected (Spearman *r* = −0.01 [*P* = .98] and *r* = −0.001 [*P* = .0996] for TRAP and CS, respectively).

**Figure 5.**
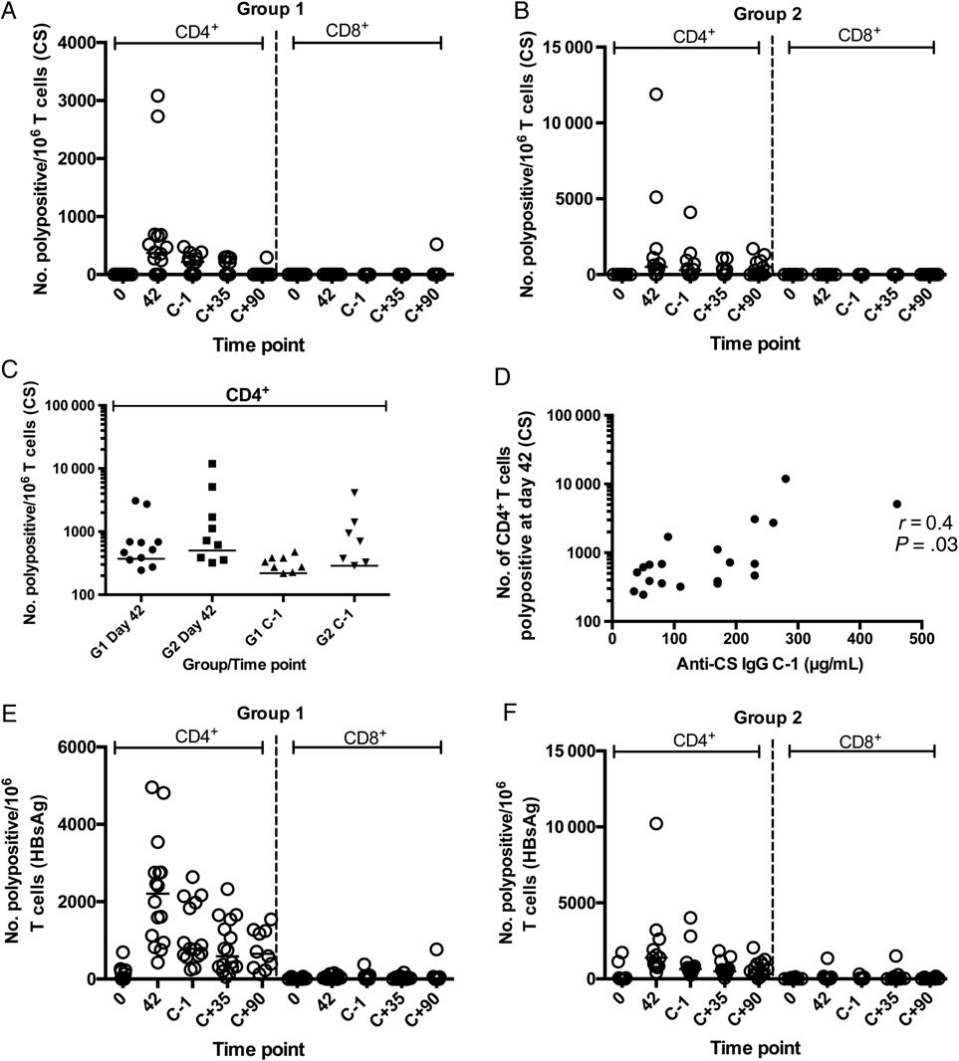
T-cell responses determined by flow cytometry on cryopreserved peripheral blood mononuclear cells before and after vaccination for circumsporozoite protein (CS) and hepatitis B virus surface antigen (HBsAg). Polypositivity indicates number of cells per million expressing ≥2 of the following markers: CD154 (CD40 ligand), interferon γ (IFN-γ), interleukin 2 (IL-2), and tumor necrosis factor α (TNF-α). *A* and *B*, Number of CS-specific polypositive CD4^+^ or CD8^+^ T cells per million in groups 1 and 2, respectively. *C*, Comparison of CD4^+^ polypositive T cells at peak time point after vaccination (day 42) and the day before controlled human malaria infection (CHMI) for groups 1 (G1) and 2 (G2). *D*, Correlation between peak CS-specific CD4^+^ polypositive frequency and anti-CS IgG level on the day before challenge (*r* = 0.4; *P* = .03, by the Spearman test). *E* and *F*, Number of HBsAg-specific polypositive CD4^+^ or CD8^+^ T cells per million in groups 1 and 2, respectively. Abbreviations: C+, elapsed time after CHMI, in days; C-1, day before CHMI; IgG, immunoglobulin G.

Flow cytometry using intracellular cytokine staining (ICS) was performed for CS and hepatitis B virus surface antigen (HBsAg) on day 42 after the first vaccination and on the day before challenge, using cryopreserved PBMCs. In this assay, responses were measured as the number of cells per million CD4^+^ or CD8^+^ T cells expressing at least 2 markers from among CD154 (CD40 ligand), IFN-γ, interleukin 2, and tumor necrosis factor α (Figure 5*A* and 5*B*). CS-specific CD4^+^ T-cell responses peaked on day 42 (2 weeks after the second dose of RTS,S) in both groups, and no significant differences were detected between groups 1 and 2 either on day 42 or the day before challenge (Figure 5*C*). CS-specific CD8^+^ T-cell responses were not detected at any significant frequency. A positive association was detected between the number of polyfunctional CD4^+^ T cells at day 42 and the level of anti-CS IgG in serum on the day before challenge (Spearman *r* = 0.4; *P* = .03; Figure 5*D*). Vaccination with RTS,S increased the frequency of HBsAg-specific CD4^+^ polyfunctional T cells in both groups at all time points after vaccination (Figure 5*E* and 5*F*).

**Figure 6.**
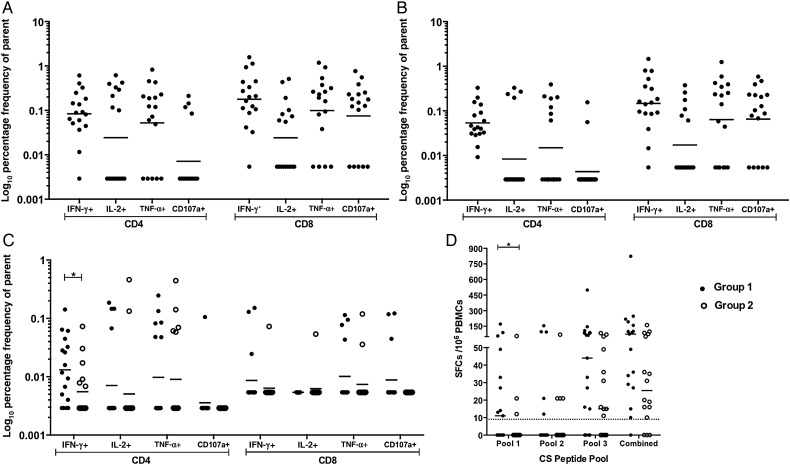
Intracellular cytokine staining of peripheral blood mononuclear cells (PBMCs) 1 day before controlled human malaria infection (CHMI; 27 days after the final RTS,S vaccination and 13 days after vaccination with modified vaccinia virus Ankara [MVA] expressing a multiepitope string fused to thrombospondin-related adhesion protein [ME-TRAP]), showing the CD107a expression frequency and the frequencies of cytokine-secreting cells as a percentage of the frequency of parent CD4^+^ and CD8^+^ T cells. Geometric mean of each response is shown in response to stimulation with TRAP T9/96 peptides (homologous to vaccine insert) by group 1 (*A*), TRAP 3D7 peptides (homologous to CHMI challenge strain) by group 1 (*B*), and circumsporozoite (CS) peptides by groups 1 and 2 (*C*). *D*, Ex vivo interferon γ enzyme-linked immunospot (ELISPOT)–determined responses of group 1 and 2 volunteers to CS peptides split into 3 peptide pools and a combined pool, with background subtracted. Dotted line shows the median background ELISPOT response, setting the positive response threshold. Data are for 17 individuals in group 1 and 16 in group 2. Data points represent individual volunteers. Abbreviations: IFN-γ, interferon γ; IL-2, interleukin 2; SFC, spot-forming cell; TNF-α, tumor necrosis factor α.

Flow cytometry was also performed on freshly isolated PBMCs, using CS peptides (for groups 1 and 2) and ME-TRAP peptides (for group 1 only) on the day before challenge. Group 1 responses to TRAP T9/96 and 3D7 were comparable across all cytokines and CD107a (*P* < .0001, by the Kruskal–Wallis test with the Dunn correction), with all volunteers exhibiting at least 1 positive cytokine response to both TRAP strains (Figure 6*A* and 6*B*). A positive response to CS was observed in 15 of 17 group 1 volunteers (83%), compared with just 9 of 16 volunteers in group 2 (56%), with a significantly higher frequency of IFN-γ–producing CD4^+^ T cells in group 1 (Figure 6*C*).

Ex vivo IFN-γ ELISPOT analysis revealed a trend toward higher responses to CS peptides in group 1 as compared to group 2 (*P* = .0517, by the Mann–Whitney test on combined groups); when assessed by peptide pool, a significant trend toward higher responses was observed in pool 1 (*P* = .0380, by the Mann–Whitney test). This is likely due to CS epitope(s) present in the ME string of ChAd63 ME-TRAP and MVA ME-TRAP. Ex vivo IFN-γ data suggest that this epitope lies toward the N-terminus of CS, as identified by a significantly higher group 1 response to peptide pool 1. The ME string contains 2 epitopes present in pool 1: CD8 epitope cp26 KPKDELDY and CD4 epitope DPNANPN, as part of a longer ME sequence, DPNANPNNVDPNANPNV (Table 1). As the main differences in ICS-determined IFN-γ production were in the CD4^+^ T-cell compartment, epitope DPNANPN could be responsible for the enhanced CS responses in group 1. This epitope is not present in RTS,S, so it was solely induced by ChAd63.MVA ME-TRAP prime boost vaccination.

**Table 1.** Comparison of Peptide Sequences Present in the Multiepitope (ME) String Fused to Thrombospondin-Related Adhesion Protein and the T-Cell Region of RTS,S

Epitope Sequence	CS Amino Acid Position (Length)	Epitope Type	Present in ME String	Present in RTS,S	Present in ELISPOT CS Peptides	ELISPOT Pool Number
DPNANPNVDP NANPNV	111–126 (16)	CD4	Yes	No	Yes, DNANPN only	1
NMPNDPN RNV	286–293 (8)	CD8	Yes	Yes	Yes, PNDPN RNV only	1
YL NKIQNSL	319–327 (9)	CD8	Yes	Yes	Yes, full length	2
KPKDELDY	353–360 (8)	CD8	Yes	Yes	Yes, full length	3

Data are from Lalvani et al [21].

Abbreviations: CS, circumsporozoite protein; ELISPOT, enzyme-linked immunospot.

## DISCUSSION

Both RTS,S/AS01_B_ and ChAd63-MVA encoding ME-TRAP have previously demonstrated partial efficacy in CHMI trials [3, 11, 12, 20], but this is the first study in which RTS,S/AS01_B_ and ChAd63-MVA ME-TRAP have been given to subjects in the same vaccine regimen. In this study, we have shown that administering these vaccines sequentially is safe, with no SUSARs and no vaccine-related serious AEs. The reactogenicity profile observed in the subjects who received the combined vaccine regimen (group 1) was similar to that observed when RTS,S/AS01_B_ or ChAd63-MVA ME-TRAP were given alone in a malaria-naive adult population [3, 11, 12, 20].

Furthermore, we have demonstrated that these vaccine candidates remain immunogenic when the regimens are combined. Anti-CS antibodies were not significantly different between group 1 and group 2 on the day before challenge, and peak numbers of TRAP-specific T cells in group 1 were similar to those observed with ChAd63-MVA ME-TRAP administered alone in a previous study [11]. GMTs of anti-CS antibodies were significantly higher in protected subjects on the day before challenge, but there was no correlation between any TRAP- or CS-specific T cell counts or TRAP-specific IgG and protection.

In this study, we observed a high level of protective efficacy in both vaccine arms. A higher proportion of subjects in group 1 remained protected following CHMI than in group 2 (82.4% vs 75%), although this difference was not statistically significant (*P* = .57). This high level of vaccine efficacy was also seen to be durable at 6 months, with 87.5% and 83.3% of initially protected subjects who underwent repeat CHMI remaining protected in groups 1 and 2, respectively. In addition, a higher proportion of subjects in group 1 reached the secondary efficacy end points of delayed time to malaria diagnosis and delayed time to PCR-confirmed parasitemia, compared with group 2. The trends observed in this study for initial challenge, rechallenge, and effects on the prepatent period are encouraging for further evaluation of the group 1 regimen, but the numbers in this study are small, and the differences observed not statistically significant. In 2013, a CHMI study of the cryopreserved whole sporozoite (PfSPZ) vaccine reported sterile efficacy of 100% in the high-dose regimen, consisting of 5 doses of 1.35 × 10^5^ parasites [22]. However, the vaccinee numbers in the high-dose group were small (n = 6), and only 5 of 6 unvaccinated controls (83.3%) developed blood-stage infection, raising concerns over the infectivity of the parasites used in that CHMI. The results observed in the trial we present in this article therefore are amongst the highest published sterile vaccine efficacy in any CHMI study in which all control subjects were infected.

The level of protective efficacy observed in the RTS,S/AS01_B_ alone group (75%) is higher than has been reported in most prior CHMI studies of this vaccine regimen [3, 20]. The mean time to patency in the control group of 12.2 days indicates that this was not an unusually weak challenge, and the vaccination and CHMI methods used in this trial are largely comparable to those in other CHMI studies of this dosing schedule of RTS,S/AS01 [3]. Practical limitations on study size are a factor for both this study and prior CHMI studies of RTS,S, resulting in a relatively small historical data set. In light of this, it is possible that the higher efficacy seen in the RTS,S alone group in this trial is a chance finding due in part to small numbers, or that further CHMI studies of RTS,S/AS01 in malaria-naive subjects, including further evaluation of differing dosing regimens and schedules, would further clarify the efficacy of the vaccine in this setting. The study was designed to have 84% power to detect a significant (*P* < .05) increase in sterile efficacy in group 1 to 90% and 69% power to detect a significant increase to 85%, compared with group 2. This power calculation assumed an expected 50% sterile efficacy in group 2 [3]. The increase in efficacy to 82.4% in group 1 from 75% in group 2 observed in this trial was not statistically significant (*P* = .69), but the power to detect a statistically significant improvement was very limited. Practical limitations of CHMI trials makes conducting large studies difficult, and designing future studies with sufficient power would be complicated, assuming an efficacy of 75% in an RTS,S/AS01_B_ alone group. One alternative approach is to wait longer after immunization, to allow vaccine efficacy to wane and thereby provide greater power to detect additive or synergist effects of combination vaccines. Further consideration of this issue and of the practical limitations of CHMI studies with challenge 3–4 weeks after the last vaccination in future trial designs is warranted.

We undertook a rechallenge of protected subjects 6 months after the initial CHMI, and 7 of 8 group 1 subjects (87.5%) and 5 of 6 group 2 subjects (83.3%) remained protected. By simply calculating the product of the percentage efficacies in the 2 CHMIs, one can estimate vaccine efficacy at 6–7 months after the immunizations as a measure of durable sterile protection. For group 1, this is 72% [(14/17) ×( 7/8) × 100], and for group 2, it is 62.5% [(12/16) × (5/6) × 100)]. Again, the durable protection rate at this time point in group 2 appears higher than in previous rechallenge trials with RTS,S/AS01_B_ administered 3 times [3], and the group 1 protection rate is even higher. This durability at 6 months is also encouraging for continued investigation of combination vaccine approaches and supports the consideration of delayed CHMI as an approach to evaluating improvements in efficacy provided by vaccines that confer substantial short-term efficacy.

In this trial, we present data from a combined vaccine regimen in which subjects received 5 vaccinations over a 10-week period. A priority for future studies should be to evaluate the effect of simplifying the vaccination schedule. A study evaluating the concomitant administration of RTS,S with viral vectors expressing ME-TRAP, thereby reducing the total number of vaccinations in a more practical schedule for potential deployment, is currently underway. These results are encouraging for further evaluation of malaria vaccine regimens that combine viral vectors with protein subunits and also vaccine regimens that target multiple stages of the malaria parasite life cycle.

## Supplementary Data

Supplementary materials are available at http://jid.oxfordjournals.org. Consisting of data provided by the author to benefit the reader, the posted materials are not copyedited and are the sole responsibility of the author, so questions or comments should be addressed to the author.

Supplementary Data
