# Supplementary material for: Safety and High Level Efficacy of the Combination Malaria Vaccine Regimen of RTS,S/AS01B With Chimpanzee Adenovirus 63 and Modified Vaccinia Ankara Vectored Vaccines Expressing ME-TRAP
Source: J Infect Dis. 2016 Jun 15;214(5):772–81. doi: 10.1093/infdis/jiw244 (PMC4978377; doi:10.1093/infdis/jiw244)
Supplement: Supplementary Data [file supp_214_5_772__index.html]

Safety and High Level Efficacy of the Combination Malaria Vaccine Regimen of RTS,S/AS01B with ChAd-MVA Vectored Vaccines Expressing ME-TRAP — Safety and High Level Efficacy of the Combination Malaria Vaccine Regimen of RTS,S/AS01B With Chimpanzee Adenovirus 63 and Modified Vaccinia Ankara Vectored Vaccines Expressing ME-TRAP — Supplementary Data 

# Safety and High Level Efficacy of the Combination Malaria Vaccine Regimen of RTS,S/AS01B With Chimpanzee Adenovirus 63 and Modified Vaccinia Ankara Vectored Vaccines Expressing ME-TRAP

## Supplementary Data

Supplementary Data

- Supplementary Data - Docx file
- Supplementary Figure 1 - docx file
- Supplementary Figure 2 - docx file
- Supplementary Table 1 - docx file
- Supplementary Table 2 - docx file
- Supplementary Table 3 - docx file
- Supplementary Table 4 - docx file
- Supplementary Table 5 - docx file
- Supplementary Table 6 - docx file
- Supplementary Table 7 - docx file
- Supplementary Table 8 - docx file
- Supplementary Table 9 - docx file
- Supplementary Table 10 - docx file
- Supplementary Table 11 - docx file
- Supplementary Table 12 - docx file
- Supplementary Table 13 - docx file
- Supplementary Table 14 - docx file
- Supplementary Table 15 - docx file
- Supplementary Table 16 - docx file
- Supplementary Table 17 - docx file
