# Supplementary material for: Safety and High Level Efficacy of the Combination Malaria Vaccine Regimen of RTS,S/AS01B With Chimpanzee Adenovirus 63 and Modified Vaccinia Ankara Vectored Vaccines Expressing ME-TRAP
Source: J Infect Dis. 2016 Jun 15;214(5):772–81. doi: 10.1093/infdis/jiw244 (PMC4978377; doi:10.1093/infdis/jiw244)
Supplement: Supplementary Data [file supp_jiw244_jiw244supp.docx]

**Safety and High Level Efficacy of the Combination Malaria Vaccine Regimen of RTS,S/AS01B with ChAd-MVA Vectored Vaccines Expressing ME-TRAP**

**Supplementary Material**

1. Supplementary Methods 2

1.1. Inclusion and Exclusion Criteria 2

1.2. Vaccines 7

1.2.1. RTS,S/AS01_B_ Vaccine 7

1.2.2. ChAd63-MVA ME-TRAP Vaccines 8

1.3. Assessment of Safety 8

1.4. *Ex-vivo* Interferon-γ (IFN-γ) Enzyme-Linked Immunosorbent Spot (ELISPOT) assays 10

1.5. Peptides for T cell Assays (Tables S9-S11) 11

1.6. Flow cytometry with Intracellular Cytokine Staining (ICS) 11

1.7. IgG ELISAs 13

1.7.1. Total IgG Enzyme Linked Immunosorbent Assay (ELISA) to TRAP 14

1.7.2. Total IgG Enzyme Linked Immunosorbent Assay (ELISA) to CS [7] 15

1.7.3. CSP-specific IgG Avidity ELISA 16

1.8. Parasite Quantitative PCR (qPCR) 17

1.9. Malaria Diagnosis 17

1.10. Parasite Growth Modelling 18

1.11. Statistical Analysis 18

1.12. Parasite density by qPCR 19

# Supplementary Methods

## Study sites

Recruitment and vaccination was conducted at three UK sites: the Centre for Clinical Vaccinology and Tropical Medicine, University of Oxford, Oxford University Hospitals NHS Trust site; the NIHR Wellcome Trust Clinical Research Facility, University of Southampton, Southampton University Hospitals NHS Trust site; and the NIHR Wellcome Trust Clinical Research Facility at the Hammersmith Hospital, Imperial College NHS Trust, London.

## Inclusion and Exclusion Criteria

**INCLUSION CRITERIA**

The volunteer must satisfy all the following criteria to be eligible for the study:

- Healthy adults aged 18 to 45 years.
- Able and willing (in the Investigator’s opinion) to comply with all study requirements.
- Willing to allow the investigators to discuss the volunteer’s medical history with their General Practitioner.
- Women only: Must practice continuous effective contraception for the duration of the study.
- Agreement to refrain from blood donation during the course of the study and for at least 3 years after the end of their involvement in the study.
- Written informed consent to participate in the trial.
- Reachable (24/7) by mobile phone during the period between CHMI and completion of antimalarial treatment.
- Willingness to take a curative anti-malaria regimen following CHMI.
- For volunteers not living in Oxford: agreement to stay in a hotel room close to the trial centre during a part of the study (from at least day 6.5 post mosquito bite until anti-malarial treatment is completed).
- Answer all questions on the informed consent quiz correctly.

**EXCLUSION CRITERIA**

The volunteer may not enter the study if any of the following apply:

- History of clinical malaria (any species).
- Travel to a malaria endemic region during the study period or within the preceding six months with significant risk of malaria exposure.
- Use of systemic antibiotics with known antimalarial activity within 30 days of CHMI (e.g. trimethoprim-sulfamethoxazole, doxycycline, tetracycline, clindamycin, erythromycin, fluoroquinolones and azithromycin)
- Receipt of an investigational product in the 30 days preceding enrolment, or planned receipt during the study period.
- Prior receipt of an investigational malaria vaccine or any other investigational vaccine likely to impact on interpretation of the trial data. If any volunteers in Group 1 and 2 undergo rechallenge, this exclusion criterion does not extend to the vaccines previously received in the VAC055 trial
- Any confirmed or suspected immunosuppressive or immunodeficient state, including HIV infection; asplenia; recurrent, severe infections and chronic (more than 14 days) immunosuppressant medication within the past 6 months (inhaled and topical steroids are allowed).
- Use of immunoglobulins or blood products within 3 months prior to enrolment.
- History of allergic disease or reactions likely to be exacerbated by any component of the vaccine (e.g. egg products, Kathon) or malaria infection.
- Any history of anaphylaxis post vaccination.
- History of clinically significant contact dermatitis.
- History of sickle cell anaemia, sickle cell trait, thalassaemia or thalassaemia trait or any haematological condition that could affect susceptibility to malaria infection.
- Pregnancy, lactation or intention to become pregnant during the study.
- Use of medications known to cause prolongation of the QT interval ***and*** existing contraindication to the use of Malarone
- Use of medications known to have a potentially clinically significant interaction with Riamet ***and*** Malarone
- Any clinical condition known to prolong the QT interval
- History of cardiac arrhythmia, including clinically relevant bradycardia
- Disturbances of electrolyte balance, eg, hypokalaemia or hypomagnesaemia
- Family history of congenital QT prolongation or sudden death
- Contraindications to the use of all three proposed anti-malarial medications; Riamet, Malarone and Chloroquine.
- History of cancer (except basal cell carcinoma of the skin and cervical carcinoma in situ).
- History of serious psychiatric condition that may affect participation in the study.
- Any other serious chronic illness requiring hospital specialist supervision.
- Suspected or known current alcohol abuse as defined by an alcohol intake of greater than 42 standard UK units every week.
- Suspected or known injecting drug abuse in the 5 years preceding enrolment.
- Hepatitis B surface antigen (HBsAg) detected in serum.
- Seropositive for hepatitis C virus (antibodies to HCV) at screening.
- An estimated, ten year risk of fatal cardiovascular disease of ≥5%, as estimated by the Systematic Coronary Risk Evaluation (SCORE) system.[^77^](#_ENREF_77)
- Positive family history in 1st and 2nd degree relatives < 50 years old for cardiac disease.
- Volunteers unable to be closely followed for social, geographic or psychological reasons.
- Any clinically significant abnormal finding on biochemistry or haematology blood tests, urinalysis or clinical examination. In the event of abnormal test results, confirmatory repeat tests will be requested. Absolute values for exclusion for confirmed abnormal results are shown in Section 17, Appendix A
- Any other significant disease, disorder, or finding which may significantly increase the risk to the volunteer because of participation in the study, affect the ability of the volunteer to participate in the study or impair interpretation of the study data.

**RE-VACCINATION EXCLUSION CRITERIA**

The following AEs associated with any vaccine, or identified on or before the day of vaccination constitute absolute contraindications to further administration of an IMP to the volunteer in question. If any of these events occur during the study, the subject will be withdrawn from the study and followed up by the clinical team or their GP until resolution or stabilisation of the event;

- Anaphylactic reaction following administration of vaccine
- Pregnancy

The following adverse events constitute contraindications to administration of vaccine at that point in time; if any one of these adverse events occurs at the time scheduled for vaccination, the subject may be vaccinated at a later date, or withdrawn, at the discretion of the investigator;

- Acute disease at the time of vaccination. (Acute disease is defined as the presence of a moderate or severe illness with or without fever.) All vaccines can be administered to persons with a minor illness such as diarrhoea, mild upper respiratory infection with or without low-grade febrile illness, *i.e.*, temperature of <37.5°C (99.5°F).
- Temperature of ≥37.5°C (99.5°F) at the time of vaccination.

Furthermore, as previously described, if any reactogenicity symptoms from the preceding RTS,S vaccination are present, the vaccination with MVA ME-TRAP will be postponed until these have resolved.

EXCLUSION CRITERION ON DAY OF CHMI

The following constitute absolute contraindications to CHMI;

- Acute disease, defined as moderate or severe illness with or without fever.
- Pregnancy.

## Vaccines

Vaccine use was authorized by the Genetically Modified Organisms Safety Committee (GMSC) of the Oxford University Hospitals NHS Trust (Reference number GM462.12.68), and the Joint Clinical Research Safety Committee of Imperial College London.

## RTS,S/AS01_B_ Vaccine

The RTS,S/AS01_B_ vaccine has been developed and manufactured by GSK Vaccines. The active substance is a recombinant antigen expressed in Saccharomyces coded RTS,S. RTS is a hybrid polypeptide consisting of a portion of the CSP antigen of the *P. falciparum* NF54 strain, fused to the amino terminal end of the hepatitis B virus surface (S) protein. S is a polypeptide corresponding to the surface antigen of hepatitis B virus (HBsAg) and is the same antigen used in GSK Vaccines’ licensed hepatitis B vaccine (Engerix-B). AS01_B_ is an Adjuvant System containing 3-*O*-desacyl-4’- monophosphoryl lipid A (MPL, 50µg, produced by GSK), *Quillaja saponaria* Molina, fraction 21 (QS-21, 50µg, Licensed by GSK from Antigenics Inc, a wholly owned subsidiary of Agenus Inc., a Delaware, USA corporation) and liposome.

## ChAd63-MVA ME-TRAP Vaccines

Both vectored vaccines in this study encoded the same insert, ME-TRAP which comprises a multiple epitope string (ME) fused to the *P. falciparum* T9/96 strain pre-erythrocytic thrombospondin-related adhesion protein (TRAP). Generation, manufacture and QC monitoring of the ChAd63 and MVA recombinant viral vectors encoding ME-TRAP has been described previously.[[1](#_ENREF_1)]

## Assessment of Safety

Safety of the IMPs was assessed by analysing the frequency, incidence and nature of adverse events and serious adverse events arising during the study.

**DEFINITIONS**

**Adverse Event (AE):** An AE is any untoward medical occurrence in a volunteer, including a dosing error, which may occur during or after administration of an IMP and does not necessarily have a causal relationship with the intervention. An AE can therefore be any unfavourable and unintended sign (including an abnormal laboratory finding), symptom or disease temporally associated with the study intervention, whether or not considered related to the study intervention.

**Adverse Reaction (AR):** An AR is any untoward or unintended response to an IMP. This means that a causal relationship between the IMP and an AE is at least a reasonable possibility, i.e., the relationship cannot be ruled out. All cases judged by either the reporting medical investigator or the sponsors as having a reasonable suspected causal relationship to an IMP (i.e. possibly, probably or definitely related to an IMP) will qualify as adverse reactions.

**Unexpected Adverse Reaction:** An adverse reaction, the nature or severity of which is not consistent with the applicable product information (*e.g.*, Investigator's Brochure for an unapproved investigational medicinal product) is considered as an unexpected adverse drug reaction.

**Serious Adverse Event (SAE):** An SAE is an AE that results in any of the following outcomes, whether or not considered related to the study intervention.

- Death (i.e., results in death from any cause at any time)
- Life-threatening event (i.e., the volunteer was, in the view of the investigator, at immediate risk of death from the event that occurred). This does not include an AE that, if it occurred in a more serious form, might have caused death.
- Persistent or significant disability or incapacity (i.e. substantial disruption of one’s ability to carry out normal life functions).
- Hospitalisation, regardless of length of stay, even if it is a precautionary measure for continued observation. Hospitalisation (including inpatient or outpatient hospitalization for an elective procedure) for a pre-existing condition that has not worsened unexpectedly does not constitute a serious AE.
- An important medical event (that may not cause death, be life threatening, or require hospitalization) that may, based upon appropriate medical judgment, jeopardize the volunteer and/or require medical or surgical intervention to prevent one of the outcomes listed above. Examples of such medical events include allergic reaction requiring intensive treatment in an emergency room or clinic, blood dyscrasias, or convulsions that do not result in inpatient hospitalization.
- Congenital anomaly or birth defect.

**Serious Adverse Reaction (SAR):** An adverse event (expected or unexpected) that is both serious and, in the opinion of the reporting investigator or sponsors, believed to be possibly, probably or definitely due to an IMP or any other study treatments, based on the information provided.

**Suspected Unexpected Serious Adverse Reactions (SUSARs):** A SUSAR is a SAE that is unexpected and thought to be possibly, probably or definitely related to an IMP.

**CAUSALITY ASSESSMENT**

For each AE, an assessment of the relationship of the AE to the study intervention(s) was undertaken. The relationship of the adverse event with the study procedures was categorized as unrelated, unlikely to be related, possibly related, probably related or definitely related. An intervention-related AE refers to an AE for which there is a possible, probable or definite relationship to the study intervention. The investigator used clinical judgment to determine the relationship. Alternative causes of the AE, such as the natural history of pre-existing medical conditions, concomitant therapy, other risk factors and the temporal relationship of the event to vaccination or CHMI was considered and investigated.

## *Ex-vivo* Interferon-γ (IFN-γ) Enzyme-Linked Immunosorbent Spot (ELISPOT) assays

*Ex vivo* (18 hour stimulation) ELISPOT assays were performed using Multiscreen IP ELISPOT plates (Millipore), human IFNγ SA-ALP antibody kits (Mabtech) and BCIP NBT-plus chromogenic substrate (Moss Inc). Cells were cultured in RPMI (Sigma) containing 1% sterile filtered Penicillin-Streptomycin (Sigma), 1% L-Glutamine (Sigma) and 10% heat-inactivated, sterile-filtered foetal calf serum, previously screened for low reactivity (Labtech International). Antigens were tested in triplicate with 250,000 PBMC added to each well of the ELISPOT plate. Plates were counted using an AID automated ELISPOT counter (AID Diagnostika GmbH, algorithm C), using identical settings for all plates and counts were adjusted only to remove artefacts. Responses to the negative control were always < 80 SFC per million PBMC. Responses were considered positive if four times greater than the negative control for the corresponding sample.

## Peptides for T cell Assays (Tables S9-S11)

TRAP peptides were 20 amino acids in length, overlapping by 10 amino acids (Neopeptide), assayed in 6 pools of 7-10 peptides at 10 μg/mL. CSP peptides were 15 amino acids in length, overlapping by 11 amino acids (a kind gift of the Malaria Department, US Naval Medical Research Center), assayed in 3 pools of 3-12 peptides at 10 μg/mL. Responses were averaged across duplicates, responses in unstimulated (negative control) wells were subtracted and then responses in individual pools were summed for each strain of the TRAP antigen or for CSP. ME responses were assayed in a single pool and peptide pool configurations are shown in Tables 14, 15, 16. Staphylococcal enterotoxin B (0.02 μg/mL) and phytohaemmagglutinin-L (10μg/mL) were used as a positive control.

## Flow cytometry with Intracellular Cytokine Staining (ICS)

Flow cytometry for vaccine-induced responses to TRAP (group 1 only) were performed at day 35 after first vaccination (D35), D77 and the day before CHMI (C-1). Flow cytometry for vaccine-induced responses to CS (group 1 and group 2) were performed at D35, D42 and C-1. Samples for flow cytometry were stimulated in parallel with the *ex-vivo* ELISPOT using fresh PBMC. After overnight stimulation, samples were stained and acquired the same day on the Jenner Institute LSR II flow cytometer. Responses to CSP and TRAP were assessed using a single pool of peptides for each antigen. Peptide sequences are described in Tables 14 and 15. Details of staining and analysis are given in Table 17. A hierarchical gating strategy was applied for analysis (Figure S1).

Responses were assessed by a 9-colour staining panel on freshly isolated PBMC, in parallel with ELISPOT assays. Aliquots of 2 × 106 PBMC in 1 ml of medium containing anti-CD28 and anti-CD49d at 1 μg ml−1 (eBioscience) and CD107a-PeCy5 (1:500, eBioscience) were stimulated with no antigen, a pool of 56 peptides spanning the T9/96 strain of the TRAP antigen (20mers overlapping by 10 amino acids, at 2 μg ml−1), a pool of 56 peptides spanning the 3D7 strain of the TRAP antigen (20mers overlapping by 10 amino acids, at 2 μg ml−1), a pool of 31 peptides spanning the CS antigen (15mers overlapping by 11 amino acids, at 2 μg ml−1) or a positive control, Staphylococcal enterotoxin B (Sigma, 1 μg ml−1) in 5 ml polystyrene FACS tubes for 18 hours at 37°C and 5% CO2. Brefeldin A and Monensin, both at 1 μg ml−1, were added for the last 16 h. Cells were incubated with a dead cell discrimination dye (AQUA 1:200, Invitrogen) for 20 minutes at room temperature. PBMC were permeabilised, then stained intracellularly at room temperature for 30 minutes with CD4-APC (1:25, eBioscience) CD14- and CD19-Pacific Blue (both 1:50, eBioscience), CD3-Alexa Fluor 700 (1:50, eBioscience), CD8-APC-Alexa Fluor 780 (1:10, eBioscience) and IFN-γ-FITC (1:100, eBioscience), IL-2-PE (1:50., eBioscience) and TNFα-Pe-Cy7 (1:500, eBioscience), then washed and fixed in 1% paraformaldehyde. Further details of monoclonal antibodies are given in Table 17. Compensation was performed using single-stained One-Comp beads (eBioscience) for monoclonal antibodies and ARC beads for AQUA (Life Technologies).

Acquisition was performed on the day of staining on a BD LSRII with median of 501,000 live CD3+ cells acquired (IQR 25% = 361750, 75% = 627000) per sample. Data was prepared and analysis performed using FlowJo v9.6.2 (Treestar Inc). Cells were gated on lymphocytes, singlets, live CD3+, CD8-CD4+ or CD4-CD8+ and then IFNγ, IL-2, TNFa and CD107a. Dead cells (AQUA+), monocytes (CD14+) and B cells (CD19+) were excluded from the analysis. All SEB stimulated PBMC gave a cytokine response >1%. Responses to peptide were determined after subtraction of the response in the unstimulated control for each sample, and considered positive if the count was >20 and frequency higher than the autologous unstimulated control and the lower limit of detection (LLD CD4+ = 0.0029, LLD CD8+ = 0.0054).

## IgG ELISAs

Anti-CS IgG was measured on serum samples collected on D0, D14, D35, D42, D70, D77, C-1, 7 days after CHMI (C+7), C+35 and C+90. For subjects who underwent repeat CHMI, further serum samples were analyzed the day before repeat CHMI (RC-1), 7 days after repeat CHMI (RC+7), RC+35 and RC+90. Antibody responses to TRAP were measured by IgG ELISA performed at the Jenner Institute, UK. Antibody responses to CS were measured by IgG ELISA performed at the WRAIR International Reference Centre for Malaria Serology.

## Total IgG Enzyme Linked Immunosorbent Assay (ELISA) to TRAP

Recombinant TRAP antigen was produced by transient transfection of HEK293E cells, using a method similar to that previously reported. [[2](#_ENREF_2), [3](#_ENREF_3)] A transgene comprising the human tissue plasminogen activator secretory signal peptide fused in frame with the 3D7-clone TRAP ectodomain (lacking the native signal peptide, transmembrane domain and a run of PNP repeats stretching from P356 to P370) was codon-optimised for mammalian expression (Life Technologies). The transgene cassette was cloned using the InFusion enzyme system (ClonTech) into the pENTR LPTOS plasmid backbone, [[4](#_ENREF_4)] in which expression of the transgene is driven by an intron-containing CMV immediate early promoter, with additional in-frame C-terminal biotin acceptor peptide and Strep(II) tags. [[5](#_ENREF_5)] Four days after polyethyleneimine mediated transfection of HEK293E cells, supernatant was harvested and affinity purified on a Streptactin affinity column (IBA Lifesciences). The resulting protein was >90% pure, as demonstrated by Coomassie Blue stained SDS-PAGE (data not shown).

Nunc-Immuno 96 well plates were coated with 0.5μg/mL of TRAP antigen in carbonate-bicarbonate coating buffer and left overnight at 4°C. Plates were washed 6x with PBS-Tween (PBS/T), then blocked with 1% BSA in PBS/T for 1 hour at room temperature (RT). Serum was diluted in PBS/T containing 0.2% BSA at concentrations of 1:100, 1:300, or 1:900, and added in triplicate. Serum samples from days 0, 28, 56, 63, C-1, C+7 and C+90 were analysed. Plates were incubated at RT for 2 hours then washed as before. A secondary antibody (goat anti-human whole IgG conjugated to alkaline phosphatase, Sigma) was added at a dilution of 1:1000 in PBS/T 0.2% BSA for 1 hour at RT. After a final wash, plates were developed by adding 4-nitrophenyl phosphate in diethanolamine buffer (Pierce).

A positive reference standard (made from pooled TRAP-positive serum) was used on each plate to give a standard curve. It was added in duplicate at an initial dilution of 1:100 (in PBS/T 0.2% BSA) and diluted 2-fold 10 times, starting with an arbitrary value of 20 antibody units. 4 blank wells (zero antibody units) were also designated. The optical density (OD) values were then fitted to a 4 parameter standard curve using SOFTmax PRO software^.^. [[6](#_ENREF_6)] An internal control was included on every plate in triplicate made up from a 1:400 dilution (in PBS/T 0.2% BSA) of the positive standard. OD was read at 405nm using an ELx800 microplate reader. Test sera antibody units were calculated from their OD values using the parameters estimated from the standard curve.

## Total IgG Enzyme Linked Immunosorbent Assay (ELISA) to CS [[7](#_ENREF_7)]

ELISA 96-well plates were coated with a synthetic peptide (Eurogentec) based on the repeat region of the PfCSP with the amino acid sequence CS(NANP)6C. The peptide was coated at a concentration of 0.2 μg/mL in a volume of 100 μL per well. Plates were placed inside a humidity chamber and incubated overnight (16 - 20 h) at 22°C. Plates were washed four times with 1xPBS (pH 7.4) containing 0.5% Tween-20 and blocked with 0.5% casein blocking buffer (Sigma) for 1 h at 22°C. Plates were washed four times and serially diluted samples were added and incubated at 22°C for 2 h. After washing four times, peroxidase labeled goat anti-human IgG (KPL) was added at a dilution of 1:4,000 and incubated at 22°C for 1 h. After washing four times, ABTS Peroxidase substrate (KPL) was added for development and incubated for 1 h at 22°C. The data were collected using Softmax Pro GXP, data were fit to a 4-parameter logistic curve, and the serum dilution at which the optical density was 1.0 (OD 1.0) calculated. To serve as a positive control, serum obtained from a volunteer participating in a Phase 1/2a challenge trial of R32NS181 formulated with alum was used. [[8](#_ENREF_8)] The individual had anti-PfCSP antibodies but was not protected (*personal communication from WRAIR serology laboratory*). Samples were considered positive if the difference between the post-immunization OD 1.0 and the pre-immunization OD 1.0 (net OD 1.0) was > 50 and the ratio of post- immunization OD 1.0 to pre-immunization OD 1.0 (ratio) was > 2. For example, if the OD 1.0 was 150 post-immunization and 50 pre-immunization, the net OD 1.0 would be 100, and the ratio of OD 1.0 post-immunization to OD 1.0 pre-immunization would be 3. This would be considered positive.

## CSP-specific IgG Avidity ELISA

IgG antibody avidity was assessed by sodium thiocyanate (NaSCN)-displacement ELISA. The assays were conducted as for total IgG ELISAs except that sera were individually diluted in casein to a level calculated to reach an OD405 of 1.0 (using total IgG EUs), and plated at 50uL/well in 16 wells of a 96 well plate. Plates were incubated for 2 hours at RT before chaotropic agent NaSCN was added in duplicate at increasing concentrations down the plate (0 to 7 Molar (M)). Plates were incubated for 15 mins at RT before washing, incubated with secondary antibody and developed with the same conditions as the total IgG assay. The concentration of NaSCN required to reduce the OD405 to 50% of that in wells where no NaSCN (=IC50) was added was used as a measure of avidity.

## Parasite Quantitative PCR (qPCR)

*P. falciparum* qPCR was performed as previously described.[[9](#_ENREF_9)] Blood was collected at baseline and at timepoints following CHMI for qPCR in 2.0ml tubes containing EDTA before being filtered to reduce white cell content. DNA was extracted from 0.5mL filtered blood using Qiagen Blood Mini Kit. 5μL of each extraction was used per assay (total eluate volume = 50μL) and was run in triplicate for qPCR (equivalent to 150μL blood directly assessed). Parasites per mL (p/mL) equivalent mean values were generated by a standard Taqman absolute quantitation, against a defined plasmid standard curve. This was conducted on an ABI StepOne Plus machine and v2.3 software using default Universal qPCR and QC settings, apart from the use of 45 cycles and 25μL reaction volume.

## Malaria Diagnosis

Diagnosis of malaria infection following CHMI was defined as positive thick film microscopy (at least one morphologically normal malaria trophozoite seen in 200 high-power (1000x) fields) by one or more experienced microscopists in a patient with symptoms suggestive of malaria.

Real time qPCR for *P. falciparum* was simultaneously performed, although Investigators (except the Chief Investigator) were blinded to the results. In the event of a positive thick film for malaria parasites in an asymptomatic volunteer, the investigators were un-blinded to the most recent PCR results for that subject only, and malaria treatment initiated only if any available PCR result for that subject had been measured as >500 parasites/ml. If all available PCR results for this subject were <500 parasites/ml, treatment was delayed until either they developed a further positive thick film in the presence of symptoms suggestive of malaria infection, or the volunteer has a further positive thick film with a PCR measurement above 500 parasites/ml.

In the event that a subject presented with symptoms or signs which were strongly suggestive of malaria infection despite having a negative blood film, investigators were un-blinded to the most recent qPCR results, and treatment initiated if any result exceeded 500 parasites/ml.

## Parasite Growth Modelling

To analyse parasite density, mean and standard deviation (SD) were calculated within each treatment group at cycle peaks (C+7.5, C+9.5 and C+11.5). For each subject the area under the curve (AUC) was calculated between first and third cycles; which was also summarized within treatment groups (mean and SD). The fold rise from cycle 1 peak was calculated at cycles 2 and 3, which was then summarized within treatment groups (mean and SD). These three characteristics of parasite density will all be compared at each time point, using an appropriate statistical method based on the distribution of the data.

## Statistical Analysis

A statistical analysis plan (SAP) was drafted by the study statistician and can be found in the supplementary appendix to this article. Data was analyzed using a variety of software including Microsoft Excel for Windows version 14 (Microsoft Corporation, Washington, USA), SPSS for Mac version 23.0 (IBM Corp., USA) and GraphPad Prism for Windows or for Mac version 6 (GraphPad Software Inc., California, USA). All tests were 2-tailed.

## Parasite density by qPCR

Parasite density was assessed by qPCR at multiple time points throughout the duration of the CHMI follow up. Results are summarized in Figure S2 and Table S13. There was a significant reduction of >95% in mean parasitaemia at the first 3 cycle peaks (C+7.5, C+9.5 and C+ 11.5) in both Group 1 and Group 2 when compared with controls (P<0.0001). In all infected subjects, parasite density at the first cycle peak (C+7.5) was negatively correlated with the time to diagnosis (Spearman R -0.576; *p*=0.039).

**SUPPLEMENTARY REFERENCES**

1. O'Hara GA, Duncan CJ, Ewer KJ, Collins KA, Elias SC, Halstead FD, et al. Clinical assessment of a recombinant simian adenovirus ChAd63: a potent new vaccine vector. The Journal of infectious diseases. 2012;205(5):772-81. Epub 2012/01/26. doi: 10.1093/infdis/jir850. PubMed PMID: 22275401; PubMed Central PMCID: PMC3274376.

2. Crosnier C, Bustamante LY, Bartholdson SJ, Bei AK, Theron M, Uchikawa M, et al. Basigin is a receptor essential for erythrocyte invasion by Plasmodium falciparum. Nature. 2011;480(7378):534-7. doi: 10.1038/nature10606. PubMed PMID: 22080952; PubMed Central PMCID: PMCPMC3245779.

3. Hodgson SH, Choudhary P, Elias SC, Milne KH, Rampling TW, Biswas S, et al. Combining viral vectored and protein-in-adjuvant vaccines against the blood-stage malaria antigen AMA1: report on a phase 1a clinical trial. Molecular therapy : the journal of the American Society of Gene Therapy. 2014;22(12):2142-54. doi: 10.1038/mt.2014.157. PubMed PMID: 25156127; PubMed Central PMCID: PMC4250079.

4. Douglas AD, Williams AR, Illingworth JJ, Kamuyu G, Biswas S, Goodman AL, et al. The blood-stage malaria antigen PfRH5 is susceptible to vaccine-inducible cross-strain neutralizing antibody. Nat Commun. 2011;2:601. Epub 2011/12/22. doi: 10.1038/ncomms1615. PubMed PMID: 22186897; PubMed Central PMCID: PMC3504505.

5. Voss S, Skerra A. Mutagenesis of a flexible loop in streptavidin leads to higher affinity for the Strep-tag II peptide and improved performance in recombinant protein purification. Protein Eng. 1997;10(8):975-82. PubMed PMID: 9415448.

6. Miura K, Orcutt AC, Muratova OV, Miller LH, Saul A, Long CA. Development and characterization of a standardized ELISA including a reference serum on each plate to detect antibodies induced by experimental malaria vaccines. Vaccine. 2008;26(2):193-200. Epub 2007/12/07. doi: 10.1016/j.vaccine.2007.10.064. PubMed PMID: 18054414; PubMed Central PMCID: PMC2253722.

7. Epstein JE, Tewari K, Lyke KE, Sim BK, Billingsley PF, Laurens MB, et al. Live attenuated malaria vaccine designed to protect through hepatic CD8(+) T cell immunity. Science. 2011;334(6055):475-80. Epub 2011/09/10. doi: 10.1126/science.1211548. PubMed PMID: 21903775.

8. Rickman LS, Gordon DM, Wistar R, Jr., Krzych U, Gross M, Hollingdale MR, et al. Use of adjuvant containing mycobacterial cell-wall skeleton, monophosphoryl lipid A, and squalane in malaria circumsporozoite protein vaccine. Lancet. 1991;337(8748):998-1001. PubMed PMID: 1673211.

9. Hodgson SH, Ewer KJ, Bliss CM, Edwards NJ, Rampling T, Anagnostou NA, et al. Evaluation of the efficacy of ChAd63-MVA vectored vaccines expressing circumsporozoite protein and ME-TRAP against controlled human malaria infection in malaria-naive individuals. The Journal of infectious diseases. 2015;211(7):1076-86. doi: 10.1093/infdis/jiu579. PubMed PMID: 25336730; PubMed Central PMCID: PMC4354983.

10. Gilbert SC, Plebanski M, Harris SJ, Allsopp CE, Thomas R, Layton GT, et al. A protein particle vaccine containing multiple malaria epitopes. Nat Biotechnol. 1997;15(12):1280-4. Epub 1997/11/14. doi: 10.1038/nbt1197-1280. PubMed PMID: 9359112.

11. Olotu A, Moris P, Mwacharo J, Vekemans J, Kimani D, Janssens M, et al. Circumsporozoite-specific T cell responses in children vaccinated with RTS,S/AS01E and protection against P falciparum clinical malaria. PLoS One. 2011;6(10):e25786. doi: 10.1371/journal.pone.0025786. PubMed PMID: 21998698; PubMed Central PMCID: PMC3188575.
