# Supplementary material for: Safety and High Level Efficacy of the Combination Malaria Vaccine Regimen of RTS,S/AS01B With Chimpanzee Adenovirus 63 and Modified Vaccinia Ankara Vectored Vaccines Expressing ME-TRAP
Source: J Infect Dis. 2016 Jun 15;214(5):772–81. doi: 10.1093/infdis/jiw244 (PMC4978377; doi:10.1093/infdis/jiw244)
Supplement: Supplementary Data [file supp_jiw244_jiw244supp_fig1.docx]

Figure S 1 Flow cytometry gating strategy. Singlets were identified using forward scatter plots. Dead cells were excluded by violet fluorescent amine-reactive dye staining. Monocytes and B cells were excluded by CD14 or CD19 expression and T cells identified by CD3 expression. T cells were then subdivided by gating on CD4+ and CD8+ populations. Cytokine expression was quantified by plotting pairs of cytokines against each other and gating positive populations. This is a representative sample from a sample stimulated overnight (18 hours) with a single pool of overlapping TRAP peptides.
