# Supplementary material for: Safety and High Level Efficacy of the Combination Malaria Vaccine Regimen of RTS,S/AS01B With Chimpanzee Adenovirus 63 and Modified Vaccinia Ankara Vectored Vaccines Expressing ME-TRAP
Source: J Infect Dis. 2016 Jun 15;214(5):772–81. doi: 10.1093/infdis/jiw244 (PMC4978377; doi:10.1093/infdis/jiw244)
Supplement: Supplementary Data [file supp_jiw244_jiw244supp_fig2.docx]

Figure S 2: A. Comparison of mean parasite densities, measured by quantitative polymerase chain reaction at cycle peaks 7.5, 9.5, and 11.5 days after controlled human malaria infection (CHMI) between vaccinees and control volunteers. Group 1 vs controls reductions of 100% (p<0.0001), 99.3% (p<0.0001) and 97.9% (p<0.0001) in mean parasite density on day 7.5, 9.5 and 11.5 respectively. Group 2 vs controls reductions of 97.4% (p<0.0001), 98.9% (p<0.0001) and 96.6% (p<0.0001) in mean parasite density on day 7.5, 9.5 and 11.5 respectively. P-values were determined by Mann-Whitney U-Test. B. Group mean log-transformed PCR data in infected volunteers. The AUC to the 3rd cycle peak (C+11.5) in infected subjects was negatively correlated with time to malaria diagnosis (Spearman R -0.711; p=0.009) C. AUC analysis of parasite densities, comparing controls to vaccinees at days 6.5–8 (the first cycle after hepatocyte release), days 8.5–10 (the second cycle), and days 10.5–12 (the third cycle) after controlled human malaria infection (CHMI). Means of log [parasite density + 1] in infected subjects were significantly different from controls in infected subjects (2-tailed t-test) in Group 1 at the first (p=0.01), second (p=0.004) and third cycles (p=0.006) and in Group 2 (p=0.02; p=0.0009 and p=0.005 at the first, second and third cycles respectively).
