# Supplementary material for: Safety and High Level Efficacy of the Combination Malaria Vaccine Regimen of RTS,S/AS01B With Chimpanzee Adenovirus 63 and Modified Vaccinia Ankara Vectored Vaccines Expressing ME-TRAP
Source: J Infect Dis. 2016 Jun 15;214(5):772–81. doi: 10.1093/infdis/jiw244 (PMC4978377; doi:10.1093/infdis/jiw244)
Supplement: Supplementary Data [file supp_jiw244_jiw244supp_table1.docx]

# Supplementary Results

## Safety - Solicited Adverse Events

| **A: Group 1 (n=20)** | |  |  |  | **B: Group 2 (n=17)** | |  |  |
| --- | --- | --- | --- | --- | --- | --- | --- | --- |
|  | **Mild (%)** | **Mod (%)** | **Sev (%)** |  |  | **Mild (%)** | **Mod (%)** | **Sev (%)** |
| Pain | 10 (50.0) | 7 (35.0) | 1 (5.0) |  | Pain | 10 (58.8) | 4 (23.5) | 1 (5.9) |
| Warmth | 9 (45.0) | 1 (5.0) | 0 (0.0) |  | Warmth | 11 (64.7) | 1 (5.9) | 0 (0.0) |
| Swelling | 2 (10.0) | 1 (5.0) | 0 (0.0) |  | Swelling | 2 (11.8) | 1 (5.9) | 0 (0.0) |
| Redness | 3 (15.0) | 0 (0.0) | 0 (0.0) |  | Redness | 2 (11.8) | 0 (0.0) | 0 (0.0) |
| Itch | 0 (0.0) | 0 (0.0) | 0 (0.0) |  | Itch | 1 (5.9) | 0 (0.0) | 0 (0.0) |
| Fever | 5 (25.0) | 1 (5.0) | 0 (0.0) |  | Fever | 1 (5.9) | 0 (0.0) | 1 (5.9) |
| Feverishness | 4 (20.0) | 5 (25.0) | 0 (0.0) |  | Feverishness | 4 (23.5) | 3 (17.6) | 1 (5.9) |
| Myalgia | 9 (45.0) | 2 (10.0) | 0 (0.0) |  | Myalgia | 2 (11.8) | 3 (17.6) | 1 (5.9) |
| Arthralgia | 7 (35.0) | 0 (0.0) | 0 (0.0) |  | Arthralgia | 2 (11.8) | 4 (23.5) | 0 (0.0) |
| Headache | 11 (55.0) | 2 (10.0) | 0 (0.0) |  | Headache | 4 (23.5) | 0 (0.0) | 1 (5.9) |
| Fatigue | 8 (40.0) | 2 (10.0) | 0 (0.0) |  | Fatigue | 2 (11.8) | 5 (29.4) | 1 (5.9) |
| Nausea | 3 (15.0) | 2 (10.0) | 0 (0.0) |  | Nausea | 1 (5.9) | 0 (0.0) | 0 (0.0) |
| Malaise | 5 (25.0) | 2 (10.0) | 0 (0.0) |  | Malaise | 4 (23.5) | 1 (5.9) | 1 (5.9) |

| **C: Group 1 (n=20)** | |  |  |  | **D: Group 2 (n=17)** | |  |  |
| --- | --- | --- | --- | --- | --- | --- | --- | --- |
|  | **Mild (%)** | **Mod (%)** | **Sev (%)** |  |  | **Mild (%)** | **Mod (%)** | **Sev (%)** |
| Pain | 16 (80.0) | 3 (15.0) | 1 (5.0) |  | Pain | 11 (64.7) | 4 (23.5) | 1 (5.9) |
| Warmth | 12 (60.0) | 1 (5.0) | 0 (0.0) |  | Warmth | 10 (58.8) | 3 (17.6) | 0 (0.0) |
| Swelling | 1 (5.0) | 0 (0.0) | 3 (15.0) |  | Swelling | 1 (5.9) | 2 (11.8) | 2 (11.8) |
| Redness | 0 (0.0) | 0 (0.0) | 0 (0.0) |  | Redness | 0 (0.0) | 0 (0.0) | 0 (0.0) |
| Itch | 2 (10.0) | 0 (0.0) | 0 (0.0) |  | Itch | 4 (23.5) | 0 (0.0) | 0 (0.0) |
| Fever | 2 (10.0) | 3 (15.0) | 0 (0.0) |  | Fever | 2 (11.8) | 3 (17.6) | 1 (5.9) |
| Feverishness | 5 (25.0) | 7 (35.0) | 2 (10.0) |  | Feverishness | 5 (29.4) | 2 (11.8) | 3 (17.6) |
| Myalgia | 8 (40.0) | 3 (15.0) | 0 (0.0) |  | Myalgia | 5 (29.4) | 4 (23.5) | 0 (0.0) |
| Arthralgia | 4 (20.0) | 1 (5.0) | 0 (0.0) |  | Arthralgia | 6 (35.3) | 3 (17.6) | 0 (0.0) |
| Headache | 10 (50.0) | 6 (30.0) | 1 (5.0) |  | Headache | 5 (29.4) | 3 (17.6) | 0 (0.0) |
| Fatigue | 11 (55.0) | 2 (10.0) | 0 (0.0) |  | Fatigue | 4 (23.5) | 4 (23.5) | 2 (11.8) |
| Nausea | 4 (20.0) | 0 (0.0) | 0 (0.0) |  | Nausea | 3 (17.6) | 2 (11.8) | 0 (0.0) |
| Malaise | 5 (25.0) | 2 (10.0) | 0 (0.0) |  | Malaise | 2 (11.8) | 5 (29.4) | 2 (11.8) |

| **E: Group 1 (n=18)** | |  |  |  | **F: Group 2 (n=16)** | |  |  |
| --- | --- | --- | --- | --- | --- | --- | --- | --- |
|  | **Mild (%)** | **Mod (%)** | **Sev (%)** |  |  | **Mild (%)** | **Mod (%)** | **Sev (%)** |
| Pain | 12 (66.7) | 5 (27.8) | 0 (0.0) |  | Pain | 12 (75.0) | 1 (6.3) | 0 (0.0) |
| Warmth | 9 (50.0) | 1 (5.6) | 0 (0.0) |  | Warmth | 11 (68.8) | 0 (0.0) | 0 (0.0) |
| Swelling | 1 (5.6) | 0 (0.0) | 2 (11.1) |  | Swelling | 4 (25.0) | 1 (6.3) | 1 (6.3) |
| Redness | 3 (16.7) | 1 (5.6) | 0 (0.0) |  | Redness | 3 (18.8) | 0 (0.0) | 0 (0.0) |
| Itch | 2 (11.1) | 0 (0.0) | 0 (0.0) |  | Itch | 1 (6.3) | 0 (0.0) | 0 (0.0) |
| Fever | 3 (16.7) | 3 (16.7) | 1 (5.6) |  | Fever | 2 (12.5) | 3 (18.8) | 0 (0.0) |
| Feverishness | 4 (22.2) | 9 (50.0) | 2 (11.1) |  | Feverishness | 5 (31.3) | 3 (18.8) | 2 (12.5) |
| Myalgia | 8 (44.4) | 4 (22.2) | 0 (0.0) |  | Myalgia | 5 (31.3) | 2 (12.5) | 0 (0.0) |
| Arthralgia | 4 (22.2) | 3 (16.7) | 1 (5.6) |  | Arthralgia | 3 (18.8) | 1 (6.3) | 0 (0.0) |
| Headache | 11 (61.1) | 3 (16.7) | 1 (5.6) |  | Headache | 4 (25.0) | 7 (43.8) | 1 (6.3) |
| Fatigue | 9 (50.0) | 3 (16.7) | 1 (5.6) |  | Fatigue | 6 (37.5) | 3 (18.8) | 0 (0.0) |
| Nausea | 3 (16.7) | 1 (5.6) | 1 (5.6) |  | Nausea | 3 (18.8) | 1 (6.3) | 0 (0.0) |
| Malaise | 5 (27.8) | 3 (16.7) | 0 (0.0) |  | Malaise | 3 (18.8) | 4 (25.0) | 1 (6.3) |

| **G: ChAd63 ME-TRAP (n=20)** | | |  |  | **H: MVA ME-TRAP (n=17)** | | |  |
| --- | --- | --- | --- | --- | --- | --- | --- | --- |
|  | **Mild (%)** | **Mod (%)** | **Sev (%)** |  |  | **Mild (%)** | **Mod (%)** | **Sev (%)** |
| Pain | 9 (45.0) | 2 (10.0) | 0 (0.0) |  | Pain | 8 (47.1) | 8 (47.1) | 1 (5.9) |
| Warmth | 6 (30.0) | 0 (0.0) | 0 (0.0) |  | Warmth | 8 (47.1) | 0 (0.0) | 0 (0.0) |
| Swelling | 1 (5.0) | 1 (5.0) | 0 (0.0) |  | Swelling | 2 (11.8) | 0 (0.0) | 0 (0.0) |
| Redness | 0 (0.0) | 1 (5.0) | 0 (0.0) |  | Redness | 5 (29.4) | 0 (0.0) | 0 (0.0) |
| Itch | 0 (0.0) | 0 (0.0) | 0 (0.0) |  | Itch | 1 (5.9) | 0 (0.0) | 0 (0.0) |
| Fever | 6 (30.0) | 0 (0.0) | 0 (0.0) |  | Fever | 6 (35.3) | 2 (11.8) | 0 (0.0) |
| Feverishness | 4 (20.0) | 6 (30.0) | 0 (0.0) |  | Feverishness | 9 (52.9) | 3 (17.6) | 3 (17.6) |
| Myalgia | 5 (25.0) | 3 (15.0) | 0 (0.0) |  | Myalgia | 6 (35.3) | 2 (11.8) | 1 (5.9) |
| Arthralgia | 5 (25.0) | 1 (5.0) | 0 (0.0) |  | Arthralgia | 7 (41.2) | 2 (11.8) | 0 (0.0) |
| Headache | 6 (30.0) | 5 (25.0) | 0 (0.0) |  | Headache | 11 (64.7) | 2 (11.8) | 0 (0.0) |
| Fatigue | 8 (40.0) | 2 (10.0) | 0 (0.0) |  | Fatigue | 12 (70.6) | 2 (11.8) | 1 (5.9) |
| Nausea | 2 (10.0) | 1 (5.0) | 0 (0.0) |  | Nausea | 5 (29.4) | 0 (0.0) | 0 (0.0) |
| Malaise | 3 (15.0) | 2 (10.0) | 0 (0.0) |  | Malaise | 5 (29.4) | 0 (0.0) | 0 (0.0) |

Table S1: A & B: Solicited adverse events reported in the 7 day period following vaccination with dose 1 of RTS,S/AS01B in A: Group 1 subjects; B: Group 2 subjects; C & D: Solicited adverse events reported in the 7 day period following vaccination with dose 2 of RTS,S/AS01B in C: Group 1 subjects; D: Group 2 subjects; E & F: Solicited adverse events reported in the 7 day period following vaccination with dose 3 of RTS,S/AS01B in E: Group 1 subjects; F: Group 2 subjects G & H: Solicited adverse events reported by Group 1 subjects in G: the 7 day period following vaccination with ChAd63 ME-TRAP; H: the 7 day period following vaccination with MVA ME-TRAP
