# Supplementary material for: Safety and High Level Efficacy of the Combination Malaria Vaccine Regimen of RTS,S/AS01B With Chimpanzee Adenovirus 63 and Modified Vaccinia Ankara Vectored Vaccines Expressing ME-TRAP
Source: J Infect Dis. 2016 Jun 15;214(5):772–81. doi: 10.1093/infdis/jiw244 (PMC4978377; doi:10.1093/infdis/jiw244)
Supplement: Supplementary Data [file supp_jiw244_jiw244supp_table2.docx]

## Safety - Unsolicited Adverse Events

| A. | RTS,S/AS01_B_ Dose 1 | | ChAd63 ME-TRAP | | RTS,S/AS01_B_ Dose 2 | | RTS,S/AS01_B_ Dose 3 | | MVA ME-TRAP | |
| --- | --- | --- | --- | --- | --- | --- | --- | --- | --- | --- |
|  | % | 95% CI | % | 95% CI | % | 95% CI | % | 95% CI | % | 95% CI |
| Group 1 | 80.0 | 56.3 - 94.3 | 75.0 | 50.9 - 91.3 | 75.0 | 50.9 - 91.3 | 66.7 | 41.0 - 86.7 | 76.5 | 50.1 - 93.2 |
| Group 2 | 70.6 | 44.0 - 89.7 |  |  | 76.5 | 50.1 - 93.2 | 50.0 | 24.7 - 75.4 |  |  |
| Total | 75.7 | 58.8 - 88.2 | 75.0 | 50.9 - 91.3 | 75.7 | 58.8 - 88.2 | 58.8 | 40.7 - 75.4 | 76.5 | 50.1 - 93.2 |

| B. | RTS,S/AS01_B_ Dose 1 | | ChAd63 ME-TRAP | | RTS,S/AS01_B_ Dose 2 | | RTS,S/AS01_B_ Dose 3 | | MVA ME-TRAP | |
| --- | --- | --- | --- | --- | --- | --- | --- | --- | --- | --- |
|  | % | 95% CI | % | 95% CI | % | 95% CI | % | 95% CI | % | 95% CI |
| Group 1 | 15.0 | 3.2 - 37.9 | 15.0 | 3.2 - 37.9 | 10.0 | 1.2 - 31.7 | 5.6 | 0.2 - 27.3 | 0.0 | 0.0 - 19.5 |
| Group 2 | 5.9 | 0.2 - 28.7 |  |  | 5.9 | 0.2 - 28.7 | 0.0 | 0.0 - 20.6 |  |  |
| Total | 10.8 | 3.0 - 25.4 | 15.0 | 3.2 - 37.9 | 8.1 | 1.7 - 22.0 | 2.9 | 0.1 - 15.3 | 0.0 | 0.0 - 19.5 |

| C. | RTS,S/AS01_B_ Dose 1 | | ChAd63 ME-TRAP | | RTS,S/AS01_B_ Dose 2 | | RTS,S/AS01_B_ Dose 3 | | MVA ME-TRAP | |
| --- | --- | --- | --- | --- | --- | --- | --- | --- | --- | --- |
|  | % | 95% CI | % | 95% CI | % | 95% CI | % | 95% CI | % | 95% CI |
| Group 1 | 25.0 | 8.7 - 49.1 | 30.0 | 11.9 - 54.3 | 35.0 | 15.4 - 59.2 | 55.6 | 30.76 - 78.47 | 35.29 | 14.21 - 61.67 |
| Group 2 | 23.5 | 6.8 - 49.9 |  |  | 35.3 | 14.2 - 61.7 | 37.5 | 15.2 - 64.57 |  |  |
| Total | 24.3 | 11.8 - 41.2 | 30.0 | 11.9 - 54.3 | 35.1 | 20.2 - 52.5 | 47.1 | 29.78 - 64.87 | 35.29 | 14.21 - 61.67 |

Table S2: A: Percentage of subjects with at least one report of unsolicited AE in the 30 day period post vaccination (Days 0-29); B: Percentage of subjects with at least one report of grade 3 unsolicited AE in the 30 day period post vaccination (Days 0-29); C: Percentage of subjects with at least one report of unsolicited AE possibly, probably or definitely related to vaccination in the 30 day period post vaccination (Days 0-29)
