# Supplementary material for: Safety and High Level Efficacy of the Combination Malaria Vaccine Regimen of RTS,S/AS01B With Chimpanzee Adenovirus 63 and Modified Vaccinia Ankara Vectored Vaccines Expressing ME-TRAP
Source: J Infect Dis. 2016 Jun 15;214(5):772–81. doi: 10.1093/infdis/jiw244 (PMC4978377; doi:10.1093/infdis/jiw244)
Supplement: Supplementary Data [file supp_jiw244_jiw244supp_table4.docx]

| **MedDRA Preferred Term (PT)** | **MedDRA Code**  **(PT)** | **Number of volunteers** | | | | **Number of occurrences** | | | |
| --- | --- | --- | --- | --- | --- | --- | --- | --- | --- |
|  |  | **Mild (%)** | **Mod (%)** | **Sev (%)** | **Total (%)** | **Mild** | **Mod** | **Sev** | **Total** |
| Abdominal pain | 10000081 | 1 (5.9) | 0 (0.0) | 0 (0.0) | 1 (5.9) | 1 | 0 | 0 | 1 |
| Blisters | 10005216 | 1 (5.9) | 0 (0.0) | 0 (0.0) | 1 (5.9) | 1 | 0 | 0 | 1 |
| Chest pain | 10008479 | 1 (5.9) | 0 (0.0) | 0 (0.0) | 1 (5.9) | 1 | 0 | 0 | 1 |
| Chills | 10008531 | 0 (0.0) | 1 (5.9) | 0 (0.0) | 1 (5.9) | 0 | 1 | 0 | 1 |
| Coryzal symptoms | 10011216 | 0 (0.0) | 1 (5.9) | 0 (0.0) | 1 (5.9) | 0 | 1 | 0 | 1 |
| Cough | 10011224 | 0 (0.0) | 1 (5.9) | 0 (0.0) | 1 (5.9) | 0 | 1 | 0 | 1 |
| Diarrhoea | 10012735 | 1 (5.9) | 0 (0.0) | 0 (0.0) | 1 (5.9) | 1 | 0 | 0 | 1 |
| Dislocation of shoulder | 10013182 | 0 (0.0) | 0 (0.0) | 1 (5.9) | 1 (5.9) | 0 | 0 | 1 | 1 |
| Exacerbation of asthma | 10015575 | 1 (5.9) | 0 (0.0) | 0 (0.0) | 1 (5.9) | 1 | 0 | 0 | 1 |
| Insomnia | 10022437 | 0 (0.0) | 2 (11.8) | 0 (0.0) | 2 (11.8) | 0 | 2 | 0 | 2 |
| Knee injury | 10049032 | 0 (0.0) | 1 (5.9) | 0 (0.0) | 1 (5.9) | 0 | 1 | 0 | 1 |
| Light headedness | 10024461 | 1 (5.9) | 0 (0.0) | 0 (0.0) | 1 (5.9) | 1 | 0 | 0 | 1 |
| Nasal congestion | 10028735 | 0 (0.0) | 1 (5.9) | 0 (0.0) | 1 (5.9) | 0 | 1 | 0 | 1 |
| Paraesthesia foot | 10052407 | 1 (5.9) | 0 (0.0) | 0 (0.0) | 1 (5.9) | 1 | 0 | 0 | 1 |
| Pharyngitis | 10034835 | 1 (5.9) | 0 (0.0) | 0 (0.0) | 1 (5.9) | 1 | 0 | 0 | 1 |
| Rectal bleeding | 10038035 | 1 (5.9) | 0 (0.0) | 0 (0.0) | 1 (5.9) | 1 | 0 | 0 | 1 |
| Rhinorrhoea | 10039101 | 1 (5.9) | 0 (0.0) | 0 (0.0) | 1 (5.9) | 1 | 0 | 0 | 1 |
| Sensation of heaviness | 10040000 | 0 (0.0) | 1 (5.9) | 0 (0.0) | 1 (5.9) | 0 | 1 | 0 | 1 |
| Urinary frequency | 10046539 | 1 (5.9) | 0 (0.0) | 0 (0.0) | 1 (5.9) | 1 | 0 | 0 | 1 |
| Wasp sting | 10047831 | 1 (5.9) | 0 (0.0) | 0 (0.0) | 1 (5.9) | 1 | 0 | 0 | 1 |

Table S4: Frequency and severity of unsolicited AEs reported by Group 2 subjects in the 30 day period following vaccination with dose 1 of RTS,S/AS01B. Proportion is performed on the per protocol cohort (n=17)
