# Supplementary material for: Safety and High Level Efficacy of the Combination Malaria Vaccine Regimen of RTS,S/AS01B With Chimpanzee Adenovirus 63 and Modified Vaccinia Ankara Vectored Vaccines Expressing ME-TRAP
Source: J Infect Dis. 2016 Jun 15;214(5):772–81. doi: 10.1093/infdis/jiw244 (PMC4978377; doi:10.1093/infdis/jiw244)
Supplement: Supplementary Data [file supp_jiw244_jiw244supp_table5.docx]

| **MedDRA Preferred Term (PT)** | **MedDRA Code**  **(PT)** | **Number of volunteers** | | | | **Number of occurrences** | | | |
| --- | --- | --- | --- | --- | --- | --- | --- | --- | --- |
|  |  | **Mild (%)** | **Mod (%)** | **Sev (%)** | **Total (%)** | **Mild** | **Mod** | **Sev** | **Total** |
| Abdominal cramps | 10000057 | 1 (5.0) | 0 (0.0) | 0 (0.0) | 1 (5.0) | 1 | 0 | 0 | 1 |
| Agitated | 10001495 | 1 (5.0) | 0 (0.0) | 0 (0.0) | 1 (5.0) | 1 | 0 | 0 | 1 |
| Allergic rhinitis | 10001723 | 1 (5.0) | 0 (0.0) | 0 (0.0) | 1 (5.0) | 1 | 0 | 0 | 1 |
| Chills | 10008531 | 0 (0.0) | 1 (5.0) | 0 (0.0) | 1 (5.0) | 0 | 1 | 0 | 1 |
| Coryzal symptoms | 10011216 | 3 (15.0) | 0 (0.0) | 1 (5.0) | 4 (20.0) | 3 | 0 | 1 | 4 |
| Cough | 10011224 | 2 (10.0) | 1 (5.0) | 0 (0.0) | 3 (15.0) | 2 | 1 | 0 | 3 |
| Diarrhoea | 10012735 | 0 (0.0) | 1 (5.0) | 0 (0.0) | 1 (5.0) | 0 | 1 | 0 | 1 |
| Dizziness | 10013573 | 0 (0.0) | 1 (5.0) | 0 (0.0) | 1 (5.0) | 0 | 1 | 0 | 1 |
| Dry cough | 10013773 | 1 (5.0) | 0 (0.0) | 0 (0.0) | 1 (5.0) | 1 | 0 | 0 | 1 |
| Dry skin | 10013786 | 1 (5.0) | 0 (0.0) | 0 (0.0) | 1 (5.0) | 1 | 0 | 0 | 1 |
| Feeling hot | 10016334 | 0 (0.0) | 1 (5.0) | 0 (0.0) | 1 (5.0) | 0 | 1 | 0 | 1 |
| Gastroenteritis | 10017888 | 0 (0.0) | 0 (0.0) | 1 (5.0) | 1 (5.0) | 0 | 0 | 1 | 1 |
| Heartburn | 10019326 | 1 (5.0) | 0 (0.0) | 0 (0.0) | 1 (5.0) | 1 | 0 | 0 | 1 |
| Hunger | 10020466 | 0 (0.0) | 1 (5.0) | 0 (0.0) | 1 (5.0) | 0 | 1 | 0 | 1 |
| Injection site bruising | 10022052 | 1 (5.0) | 0 (0.0) | 0 (0.0) | 1 (5.0) | 1 | 0 | 0 | 1 |
| Insomnia | 10022437 | 1 (5.0) | 0 (0.0) | 0 (0.0) | 1 (5.0) | 1 | 0 | 0 | 1 |
| Laryngitis | 10023874 | 1 (5.0) | 0 (0.0) | 0 (0.0) | 1 (5.0) | 1 | 0 | 0 | 1 |
| Light headed feeling | 10024491 | 1 (5.0) | 0 (0.0) | 0 (0.0) | 1 (5.0) | 1 | 0 | 0 | 1 |
| Localized erythema | 10024782 | 1 (5.0) | 0 (0.0) | 0 (0.0) | 1 (5.0) | 1 | 0 | 0 | 1 |
| Localized superficial swelling, mass or lump | 10024784 | 1 (5.0) | 0 (0.0) | 0 (0.0) | 1 (5.0) | 1 | 0 | 0 | 1 |
| Lymphadenopathy cervical | 10025200 | 1 (5.0) | 0 (0.0) | 0 (0.0) | 1 (5.0) | 1 | 0 | 0 | 1 |
| Middle ear effusion | 10062545 | 1 (5.0) | 0 (0.0) | 0 (0.0) | 1 (5.0) | 1 | 0 | 0 | 1 |
| Muscle cramp | 10028294 | 0 (0.0) | 0 (0.0) | 1 (5.0) | 1 (5.0) | 0 | 0 | 1 | 1 |
| Nasal congestion | 10028735 | 1 (5.0) | 0 (0.0) | 0 (0.0) | 1 (5.0) | 1 | 0 | 0 | 1 |
| Period pains | 10034532 | 1 (5.0) | 0 (0.0) | 0 (0.0) | 1 (5.0) | 1 | 0 | 0 | 1 |
| Pharyngitis | 10034835 | 3 (15.0) | 0 (0.0) | 0 (0.0) | 3 (15.0) | 3 | 0 | 0 | 3 |
| Red throat | 10038196 | 1 (5.0) | 0 (0.0) | 0 (0.0) | 1 (5.0) | 1 | 0 | 0 | 1 |
| Sensation of warmth | 10040006 | 1 (5.0) | 1 (5.0) | 0 (0.0) | 2 (10.0) | 1 | 1 | 0 | 2 |
| Sneezing | 10041232 | 1 (5.0) | 1 (5.0) | 0 (0.0) | 2 (10.0) | 2 | 1 | 0 | 3 |
| Subjective visual disturbance, unspecified | 10042399 | 0 (0.0) | 1 (5.0) | 0 (0.0) | 1 (5.0) | 0 | 1 | 0 | 1 |
| Thumb sprain | 10043659 | 1 (5.0) | 0 (0.0) | 0 (0.0) | 1 (5.0) | 1 | 0 | 0 | 1 |
| Tight chest | 10043854 | 0 (0.0) | 1 (5.0) | 0 (0.0) | 1 (5.0) | 0 | 1 | 0 | 1 |
| Toothache | 10044057 | 0 (0.0) | 1 (5.0) | 0 (0.0) | 1 (5.0) | 0 | 1 | 0 | 1 |

Table S5: Frequency and severity of unsolicited AEs reported by Group 1 subjects in the 30 day period following vaccination with ChAd63 ME-TRAP. Proportion is performed on the per protocol cohort (n=17)
