# Supplementary material for: Safety and High Level Efficacy of the Combination Malaria Vaccine Regimen of RTS,S/AS01B With Chimpanzee Adenovirus 63 and Modified Vaccinia Ankara Vectored Vaccines Expressing ME-TRAP
Source: J Infect Dis. 2016 Jun 15;214(5):772–81. doi: 10.1093/infdis/jiw244 (PMC4978377; doi:10.1093/infdis/jiw244)
Supplement: Supplementary Data [file supp_jiw244_jiw244supp_table6.docx]

| **MedDRA Preferred Term (PT)** | **MedDRA Code**  **(PT)** | **Number of volunteers** | | | | **Number of occurrences** | | | |
| --- | --- | --- | --- | --- | --- | --- | --- | --- | --- |
|  |  | **Mild (%)** | **Mod (%)** | **Sev (%)** | **Total (%)** | **Mild** | **Mod** | **Sev** | **Total** |
| Agitated | 10001495 | 1 (5.0) | 0 (0.0) | 0 (0.0) | 1 (5.0) | 1 | 0 | 0 | 1 |
| Chills | 10008531 | 1 (5.0) | 0 (0.0) | 0 (0.0) | 1 (5.0) | 1 | 0 | 0 | 1 |
| Coryzal symptoms | 10011216 | 3 (15.0) | 1 (5.0) | 0 (0.0) | 4 (20.0) | 4 | 1 | 0 | 5 |
| Cough | 10011224 | 1 (5.0) | 1 (5.0) | 0 (0.0) | 2 (10.0) | 1 | 1 | 0 | 2 |
| Diarrhoea | 10012735 | 0 (0.0) | 1 (5.0) | 0 (0.0) | 1 (5.0) | 0 | 1 | 0 | 1 |
| Dizziness | 10013573 | 0 (0.0) | 1 (5.0) | 0 (0.0) | 1 (5.0) | 0 | 1 | 0 | 1 |
| Dry cough | 10013773 | 1 (5.0) | 0 (0.0) | 0 (0.0) | 1 (5.0) | 1 | 0 | 0 | 1 |
| Dry skin | 10013786 | 1 (5.0) | 0 (0.0) | 0 (0.0) | 1 (5.0) | 1 | 0 | 0 | 1 |
| Feeling hot | 10016334 | 0 (0.0) | 1 (5.0) | 0 (0.0) | 1 (5.0) | 0 | 1 | 0 | 1 |
| Heartburn | 10019326 | 1 (5.0) | 0 (0.0) | 0 (0.0) | 1 (5.0) | 1 | 0 | 0 | 1 |
| Indigestion | 10021706 | 1 (5.0) | 0 (0.0) | 0 (0.0) | 1 (5.0) | 1 | 0 | 0 | 1 |
| Insomnia | 10022437 | 1 (5.0) | 0 (0.0) | 0 (0.0) | 1 (5.0) | 1 | 0 | 0 | 1 |
| Knee pain | 10023477 | 0 (0.0) | 1 (5.0) | 0 (0.0) | 1 (5.0) | 0 | 1 | 0 | 1 |
| Laryngitis | 10023874 | 1 (5.0) | 0 (0.0) | 0 (0.0) | 1 (5.0) | 1 | 0 | 0 | 1 |
| Localized erythema | 10024782 | 1 (5.0) | 0 (0.0) | 0 (0.0) | 1 (5.0) | 2 | 0 | 0 | 2 |
| Loose bowels | 10024838 | 0 (0.0) | 1 (5.0) | 0 (0.0) | 1 (5.0) | 0 | 1 | 0 | 1 |
| Low back pain | 10024891 | 1 (5.0) | 0 (0.0) | 1 (5.0) | 2 (10.0) | 1 | 0 | 1 | 2 |
| Lymphadenopathy cervical | 10025200 | 1 (5.0) | 0 (0.0) | 0 (0.0) | 1 (5.0) | 1 | 0 | 0 | 1 |
| Middle ear effusion | 10062545 | 1 (5.0) | 0 (0.0) | 0 (0.0) | 1 (5.0) | 1 | 0 | 0 | 1 |
| Muscle cramp | 10028294 | 0 (0.0) | 0 (0.0) | 1 (5.0) | 1 (5.0) | 0 | 0 | 1 | 1 |
| Nasal congestion | 10028735 | 1 (5.0) | 0 (0.0) | 0 (0.0) | 1 (5.0) | 1 | 0 | 0 | 1 |
| Neck pain | 10028836 | 0 (0.0) | 1 (5.0) | 0 (0.0) | 1 (5.0) | 0 | 1 | 0 | 1 |
| Night sweats | 10029410 | 2 (10.0) | 0 (0.0) | 0 (0.0) | 2 (10.0) | 2 | 0 | 0 | 2 |
| Pharyngitis | 10034835 | 2 (10.0) | 0 (0.0) | 0 (0.0) | 2 (10.0) | 2 | 0 | 0 | 2 |
| Pulled hamstring | 10037305 | 1 (5.0) | 0 (0.0) | 0 (0.0) | 1 (5.0) | 1 | 0 | 0 | 1 |
| Red throat | 10038196 | 1 (5.0) | 0 (0.0) | 0 (0.0) | 1 (5.0) | 1 | 0 | 0 | 1 |
| Sensation of warmth | 10040006 | 1 (5.0) | 1 (5.0) | 0 (0.0) | 2 (10.0) | 1 | 1 | 0 | 2 |
| Sneezing | 10041232 | 1 (5.0) | 1 (5.0) | 0 (0.0) | 2 (10.0) | 1 | 1 | 0 | 2 |
| Subjective visual disturbance, unspecified | 10042399 | 0 (0.0) | 1 (5.0) | 0 (0.0) | 1 (5.0) | 0 | 1 | 0 | 1 |
| Swelling, mass or lump in head and neck | 10042710 | 1 (5.0) | 0 (0.0) | 0 (0.0) | 1 (5.0) | 1 | 0 | 0 | 1 |
| Tight chest | 10043854 | 0 (0.0) | 1 (5.0) | 0 (0.0) | 1 (5.0) | 0 | 1 | 0 | 1 |
| Toothache | 10044055 | 0 (0.0) | 1 (5.0) | 0 (0.0) | 1 (5.0) | 0 | 1 | 0 | 1 |

Table S6: Frequency and severity of unsolicited AEs reported by Group 1 subjects in the 30 day period following vaccination with dose 2 of RTS,S/AS01B. Proportion is performed on the per protocol cohort (n=20)
