# Supplementary material for: Safety and High Level Efficacy of the Combination Malaria Vaccine Regimen of RTS,S/AS01B With Chimpanzee Adenovirus 63 and Modified Vaccinia Ankara Vectored Vaccines Expressing ME-TRAP
Source: J Infect Dis. 2016 Jun 15;214(5):772–81. doi: 10.1093/infdis/jiw244 (PMC4978377; doi:10.1093/infdis/jiw244)
Supplement: Supplementary Data [file supp_jiw244_jiw244supp_table7.docx]

| **MedDRA Preferred Term (PT)** | **MedDRA Code**  **(PT)** | **Number of volunteers** | | | | **Number of occurrences** | | | |
| --- | --- | --- | --- | --- | --- | --- | --- | --- | --- |
|  |  | **Mild (%)** | **Mod (%)** | **Sev (%)** | **Total (%)** | **Mild** | **Mod** | **Sev** | **Total** |
| Abdominal pain | 10000081 | 2 (11.8) | 0 (0.0) | 0 (0.0) | 2 (11.8) | 3 | 0 | 0 | 3 |
| Asthmatic wheezing | 10049200 | 1 (5.9) | 0 (0.0) | 0 (0.0) | 1 (5.9) | 1 | 0 | 0 | 1 |
| Backache | 10003993 | 1 (5.9) | 0 (0.0) | 0 (0.0) | 1 (5.9) | 1 | 0 | 0 | 1 |
| Chest pain | 10008479 | 1 (5.9) | 0 (0.0) | 0 (0.0) | 1 (5.9) | 1 | 0 | 0 | 1 |
| Chills | 10008531 | 0 (0.0) | 1 (5.9) | 0 (0.0) | 1 (5.9) | 0 | 1 | 0 | 1 |
| Constipation | 10010774 | 1 (5.9) | 0 (0.0) | 0 (0.0) | 1 (5.9) | 1 | 0 | 0 | 1 |
| Coryzal symptoms | 10011216 | 2 (11.8) | 0 (0.0) | 0 (0.0) | 2 (11.8) | 2 | 0 | 0 | 2 |
| Cough | 10011224 | 0 (0.0) | 1 (5.9) | 0 (0.0) | 1 (5.9) | 0 | 1 | 0 | 1 |
| Diarrhoea | 10012735 | 0 (0.0) | 2 (11.8) | 0 (0.0) | 2 (11.8) | 0 | 2 | 0 | 2 |
| Dislocation of shoulder | 10013182 | 0 (0.0) | 0 (0.0) | 1 (5.9) | 1 (5.9) | 0 | 0 | 1 | 1 |
| Eye pain | 10015958 | 1 (5.9) | 0 (0.0) | 0 (0.0) | 1 (5.9) | 1 | 0 | 0 | 1 |
| Head injury | 10019196 | 1 (5.9) | 0 (0.0) | 0 (0.0) | 1 (5.9) | 1 | 0 | 0 | 1 |
| Insomnia | 10022437 | 0 (0.0) | 1 (5.9) | 0 (0.0) | 1 (5.9) | 0 | 1 | 0 | 1 |
| Knee pain | 10023477 | 1 (5.9) | 0 (0.0) | 0 (0.0) | 1 (5.9) | 1 | 0 | 0 | 1 |
| Menstrual cycle shortened | 10027326 | 1 (5.9) | 0 (0.0) | 0 (0.0) | 1 (5.9) | 1 | 0 | 0 | 1 |
| Migraine | 10027599 | 0 (0.0) | 1 (5.9) | 0 (0.0) | 1 (5.9) | 0 | 1 | 0 | 1 |
| Night sweats | 10029410 | 0 (0.0) | 1 (5.9) | 0 (0.0) | 1 (5.9) | 0 | 1 | 0 | 1 |
| Rectal bleeding | 10038035 | 1 (5.9) | 0 (0.0) | 0 (0.0) | 1 (5.9) | 1 | 0 | 0 | 1 |
| Shooting pain | 10050007 | 0 (0.0) | 1 (5.9) | 0 (0.0) | 1 (5.9) | 0 | 1 | 0 | 1 |
| Sore throat | 10041367 | 1 (5.9) | 0 (0.0) | 0 (0.0) | 1 (5.9) | 1 | 0 | 0 | 1 |
| Subjective visual disturbance, unspecified | 10042399 | 1 (5.9) | 0 (0.0) | 0 (0.0) | 1 (5.9) | 1 | 0 | 0 | 1 |
| Sweating | 10042661 | 1 (5.9) | 0 (0.0) | 0 (0.0) | 1 (5.9) | 1 | 0 | 0 | 1 |
| Vomiting | 10047700 | 1 (5.9) | 1 (5.9) | 0 (0.0) | 2 (11.8) | 1 | 1 | 0 | 2 |
| Wasp sting | 10047831 | 1 (5.9) | 0 (0.0) | 0 (0.0) | 1 (5.9) | 1 | 0 | 0 | 1 |
| Wrist pain | 10048692 | 1 (5.9) | 0 (0.0) | 0 (0.0) | 1 (5.9) | 1 | 0 | 0 | 1 |

Table S7: Frequency and severity of unsolicited AEs reported by Group 2 subjects in the 30 day period following vaccination with dose 2 of RTS,S/AS01B. Proportion is performed on the per protocol cohort (n=17)
