# Supplementary material for: Safety and High Level Efficacy of the Combination Malaria Vaccine Regimen of RTS,S/AS01B With Chimpanzee Adenovirus 63 and Modified Vaccinia Ankara Vectored Vaccines Expressing ME-TRAP
Source: J Infect Dis. 2016 Jun 15;214(5):772–81. doi: 10.1093/infdis/jiw244 (PMC4978377; doi:10.1093/infdis/jiw244)
Supplement: Supplementary Data [file supp_jiw244_jiw244supp_table8.docx]

| **MedDRA Preferred Term (PT)** | **MedDRA Code**  **(PT)** | **Number of volunteers** | | | | **Number of occurrences** | | | |
| --- | --- | --- | --- | --- | --- | --- | --- | --- | --- |
|  |  | **Mild (%)** | **Mod (%)** | **Sev (%)** | **Total (%)** | **Mild** | **Mod** | **Sev** | **Total** |
| Allergic skin reaction | 10001729 | 1 (5.6) | 0 (0.0) | 0 (0.0) | 1 (5.6) | 1 | 0 | 0 | 1 |
| Backache | 10003993 | 1 (5.6) | 0 (0.0) | 0 (0.0) | 1 (5.6) | 1 | 0 | 0 | 1 |
| Chills | 10008531 | 1 (5.6) | 1 (5.6) | 0 (0.0) | 2 (11.1) | 1 | 1 | 0 | 2 |
| Coryzal symptoms | 10011216 | 1 (5.6) | 0 (0.0) | 0 (0.0) | 1 (5.6) | 1 | 0 | 0 | 1 |
| Eyelid rash | 10074620 | 1 (5.6) | 0 (0.0) | 0 (0.0) | 1 (5.6) | 1 | 0 | 0 | 1 |
| Indigestion | 10021706 | 1 (5.6) | 0 (0.0) | 0 (0.0) | 1 (5.6) | 1 | 0 | 0 | 1 |
| Insomnia | 10022437 | 0 (0.0) | 1 (5.6) | 0 (0.0) | 1 (5.6) | 0 | 1 | 0 | 1 |
| Knee pain | 10023477 | 0 (0.0) | 1 (5.6) | 0 (0.0) | 1 (5.6) | 0 | 1 | 0 | 1 |
| Light headedness | 10024461 | 1 (5.6) | 0 (0.0) | 0 (0.0) | 1 (5.6) | 1 | 0 | 0 | 1 |
| Localized erythema | 10024781 | 1 (5.6) | 0 (0.0) | 0 (0.0) | 1 (5.6) | 1 | 0 | 0 | 1 |
| Loose bowels | 10024838 | 0 (0.0) | 1 (5.6) | 0 (0.0) | 1 (5.6) | 0 | 1 | 0 | 1 |
| Low back pain | 10024891 | 2 (11.1) | 0 (0.0) | 0 (0.0) | 2 (11.1) | 2 | 0 | 0 | 2 |
| Nasal congestion | 10028735 | 1 (5.6) | 0 (0.0) | 0 (0.0) | 1 (5.6) | 1 | 0 | 0 | 1 |
| Neck pain | 10028836 | 0 (0.0) | 1 (5.6) | 0 (0.0) | 1 (5.6) | 0 | 1 | 0 | 1 |
| Night sweats | 10029410 | 2 (11.1) | 0 (0.0) | 0 (0.0) | 2 (11.1) | 2 | 0 | 0 | 2 |
| Pain in arm | 10033421 | 0 (0.0) | 0 (0.0) | 1 (5.6) | 1 (5.6) | 0 | 0 | 1 | 1 |
| Pharyngitis | 10034835 | 2 (11.1) | 0 (0.0) | 0 (0.0) | 2 (11.1) | 2 | 0 | 0 | 2 |
| Polydipsia | 10036067 | 1 (5.6) | 0 (0.0) | 0 (0.0) | 1 (5.6) | 1 | 0 | 0 | 1 |
| Rash erythematous | 10037855 | 1 (5.6) | 0 (0.0) | 0 (0.0) | 1 (5.6) | 1 | 0 | 0 | 1 |
| Sensation of cold | 10039997 | 0 (0.0) | 1 (5.6) | 0 (0.0) | 1 (5.6) | 0 | 1 | 0 | 1 |
| Sore throat | 10041367 | 1 (5.6) | 0 (0.0) | 0 (0.0) | 1 (5.6) | 1 | 0 | 0 | 1 |
| Sweating fever | 10042666 | 0 (0.0) | 1 (5.6) | 0 (0.0) | 1 (5.6) | 0 | 1 | 0 | 1 |
| Swelling, mass or lump in head and neck | 10042710 | 1 (5.6) | 0 (0.0) | 0 (0.0) | 1 (5.6) | 1 | 0 | 0 | 1 |
| Toothache | 10044055 | 0 (0.0) | 1 (5.6) | 0 (0.0) | 1 (5.6) | 0 | 1 | 0 | 1 |

Table S8: Frequency and severity of unsolicited AEs reported by Group 1 subjects in the 30 day period following vaccination with dose 3 of RTS,S/AS01B. Proportion is performed on the per protocol cohort (n=18)
