# Supplementary material for: Safety and High Level Efficacy of the Combination Malaria Vaccine Regimen of RTS,S/AS01B With Chimpanzee Adenovirus 63 and Modified Vaccinia Ankara Vectored Vaccines Expressing ME-TRAP
Source: J Infect Dis. 2016 Jun 15;214(5):772–81. doi: 10.1093/infdis/jiw244 (PMC4978377; doi:10.1093/infdis/jiw244)
Supplement: Supplementary Data [file supp_jiw244_jiw244supp_table9.docx]

| **MedDRA Preferred Term (PT)** | **MedDRA Code**  **(PT)** | **Number of volunteers** | | | | **Number of occurrences** | | | |
| --- | --- | --- | --- | --- | --- | --- | --- | --- | --- |
|  |  | **Mild (%)** | **Mod (%)** | **Sev (%)** | **Total (%)** | **Mild** | **Mod** | **Sev** | **Total** |
| Abdominal pain | 10000081 | 1 (6.3) | 0 (0.0) | 0 (0.0) | 1 (6.3) | 1 | 0 | 0 | 1 |
| Backache | 10003993 | 1 (6.3) | 0 (0.0) | 0 (0.0) | 1 (6.3) | 1 | 0 | 0 | 1 |
| Coryzal symptoms | 10011216 | 2 (12.5) | 0 (0.0) | 0 (0.0) | 2 (12.5) | 2 | 0 | 0 | 2 |
| Cough | 10011224 | 1 (6.3) | 0 (0.0) | 0 (0.0) | 1 (6.3) | 1 | 0 | 0 | 1 |
| Eye pain | 10015958 | 1 (6.3) | 0 (0.0) | 0 (0.0) | 1 (6.3) | 1 | 0 | 0 | 1 |
| Head injury | 10019196 | 1 (6.3) | 0 (0.0) | 0 (0.0) | 1 (6.3) | 1 | 0 | 0 | 1 |
| Local swelling | 10024770 | 1 (6.3) | 0 (0.0) | 0 (0.0) | 1 (6.3) | 1 | 0 | 0 | 1 |
| Night sweats | 10029410 | 0 (0.0) | 1 (6.3) | 0 (0.0) | 1 (6.3) | 0 | 1 | 0 | 1 |
| Period pains | 10034532 | 1 (6.3) | 0 (0.0) | 0 (0.0) | 1 (6.3) | 1 | 0 | 0 | 1 |
| Rash over arms | 10037875 | 1 (6.3) | 0 (0.0) | 0 (0.0) | 1 (6.3) | 1 | 0 | 0 | 1 |
| Subjective visual disturbance, unspecified | 10042399 | 1 (6.3) | 0 (0.0) | 0 (0.0) | 1 (6.3) | 1 | 0 | 0 | 1 |
| Sweating | 10042661 | 1 (6.3) | 0 (0.0) | 0 (0.0) | 1 (6.3) | 1 | 0 | 0 | 1 |

Table S9: Frequency and severity of unsolicited AEs reported by Group 2 subjects in the 30 day period following vaccination with dose 3 of RTS,S/AS01B. Proportion is performed on the per protocol cohort (n=16)
