# Supplementary material for: Safety and High Level Efficacy of the Combination Malaria Vaccine Regimen of RTS,S/AS01B With Chimpanzee Adenovirus 63 and Modified Vaccinia Ankara Vectored Vaccines Expressing ME-TRAP
Source: J Infect Dis. 2016 Jun 15;214(5):772–81. doi: 10.1093/infdis/jiw244 (PMC4978377; doi:10.1093/infdis/jiw244)
Supplement: Supplementary Data [file supp_jiw244_jiw244supp_table10.docx]

| **MedDRA Preferred Term (PT)** | **MedDRA Code**  **(PT)** | **Number of volunteers** | | | | **Number of occurrences** | | | |
| --- | --- | --- | --- | --- | --- | --- | --- | --- | --- |
|  |  | **Mild (%)** | **Mod (%)** | **Sev (%)** | **Total (%)** | **Mild** | **Mod** | **Sev** | **Total** |
| Abdominal cramps | 10000057 | 1 (5.9) | 0 (0.0) | 0 (0.0) | 1 (5.9) | 1 | 0 | 0 | 1 |
| Allergic skin reaction | 10001729 | 1 (5.9) | 0 (0.0) | 0 (0.0) | 1 (5.9) | 1 | 0 | 0 | 1 |
| Athlete's foot | 10003621 | 1 (5.9) | 0 (0.0) | 0 (0.0) | 1 (5.9) | 1 | 0 | 0 | 1 |
| Backache | 10003993 | 1 (5.9) | 0 (0.0) | 0 (0.0) | 1 (5.9) | 1 | 0 | 0 | 1 |
| Buttock pain | 10048677 | 1 (5.9) | 0 (0.0) | 0 (0.0) | 1 (5.9) | 1 | 0 | 0 | 1 |
| Chills | 10008531 | 0 (0.0) | 1 (5.9) | 0 (0.0) | 1 (5.9) | 0 | 1 | 0 | 1 |
| Coryzal symptoms | 10011216 | 1 (5.9) | 0 (0.0) | 0 (0.0) | 1 (5.9) | 1 | 0 | 0 | 1 |
| Cough | 10011224 | 0 (0.0) | 1 (5.9) | 0 (0.0) | 1 (5.9) | 0 | 1 | 0 | 1 |
| Dizziness | 10013573 | 1 (5.9) | 0 (0.0) | 0 (0.0) | 1 (5.9) | 1 | 0 | 0 | 1 |
| Epistaxis | 10015090 | 1 (5.9) | 0 (0.0) | 0 (0.0) | 1 (5.9) | 1 | 0 | 0 | 1 |
| Eye pain | 10015958 | 1 (5.9) | 0 (0.0) | 0 (0.0) | 1 (5.9) | 1 | 0 | 0 | 1 |
| Eyelid rash | 10074620 | 1 (5.9) | 0 (0.0) | 0 (0.0) | 1 (5.9) | 1 | 0 | 0 | 1 |
| Head injury | 10019196 | 1 (5.9) | 0 (0.0) | 0 (0.0) | 1 (5.9) | 1 | 0 | 0 | 1 |
| Insect bite NOS | 10022403 | 1 (5.9) | 0 (0.0) | 0 (0.0) | 1 (5.9) | 1 | 0 | 0 | 1 |
| Insomnia | 10022437 | 0 (0.0) | 1 (5.9) | 0 (0.0) | 1 (5.9) | 0 | 1 | 0 | 1 |
| Itching both hands | 10023087 | 0 (0.0) | 1 (5.9) | 0 (0.0) | 1 (5.9) | 0 | 1 | 0 | 1 |
| Light headedness | 10024461 | 1 (5.9) | 1 (5.9) | 0 (0.0) | 2 (11.8) | 1 | 1 | 0 | 2 |
| Low back pain | 10024891 | 1 (5.9) | 0 (0.0) | 0 (0.0) | 1 (5.9) | 1 | 0 | 0 | 1 |
| Nasal congestion | 10028735 | 1 (5.9) | 0 (0.0) | 0 (0.0) | 1 (5.9) | 1 | 0 | 0 | 1 |
| Neck pain | 10028836 | 1 (5.9) | 0 (0.0) | 0 (0.0) | 1 (5.9) | 1 | 0 | 0 | 1 |
| Pain in ankle | 10033420 | 1 (5.9) | 0 (0.0) | 0 (0.0) | 1 (5.9) | 1 | 0 | 0 | 1 |
| Pain in leg | 10033446 | 0 (0.0) | 1 (5.9) | 0 (0.0) | 1 (5.9) | 0 | 1 | 0 | 1 |
| Pain in thigh | 10048973 | 1 (5.9) | 0 (0.0) | 0 (0.0) | 1 (5.9) | 1 | 0 | 0 | 1 |
| Palpitations | 10033557 | 1 (5.9) | 0 (0.0) | 0 (0.0) | 1 (5.9) | 1 | 0 | 0 | 1 |
| Period pains | 10034532 | 1 (5.9) | 0 (0.0) | 0 (0.0) | 1 (5.9) | 1 | 0 | 0 | 1 |
| Pharyngitis | 10034835 | 1 (5.9) | 1 (5.9) | 0 (0.0) | 2 (11.8) | 2 | 1 | 0 | 3 |
| Polydipsia | 10036067 | 1 (5.9) | 0 (0.0) | 0 (0.0) | 1 (5.9) | 1 | 0 | 0 | 1 |
| Pruritis | 10037086 | 1 (5.9) | 0 (0.0) | 0 (0.0) | 1 (5.9) | 1 | 0 | 0 | 1 |
| Rash erythematous | 10037855 | 1 (5.9) | 0 (0.0) | 0 (0.0) | 1 (5.9) | 1 | 0 | 0 | 1 |
| Rhinorrhoea | 10039101 | 0 (0.0) | 1 (5.9) | 0 (0.0) | 1 (5.9) | 0 | 1 | 0 | 1 |
| Sensation of cold | 10039997 | 0 (0.0) | 1 (5.9) | 0 (0.0) | 1 (5.9) | 0 | 1 | 0 | 1 |
| Sensation of warmth | 10040006 | 1 (5.9) | 0 (0.0) | 0 (0.0) | 1 (5.9) | 1 | 0 | 0 | 1 |
| Toe injury | 10051023 | 1 (5.9) | 0 (0.0) | 0 (0.0) | 1 (5.9) | 1 | 0 | 0 | 1 |
| Toothache | 10044055 | 1 (5.9) | 0 (0.0) | 0 (0.0) | 1 (5.9) | 1 | 0 | 0 | 1 |
| Twitching | 10045198 | 1 (5.9) | 0 (0.0) | 0 (0.0) | 1 (5.9) | 1 | 0 | 0 | 1 |
| Urinary frequency | 10046539 | 1 (5.9) | 0 (0.0) | 0 (0.0) | 1 (5.9) | 1 | 0 | 0 | 1 |

Table S10: Frequency and severity of unsolicited AEs reported by Group 1 subjects in the 30 day period following vaccination with MVA ME-TRAP. Proportion is performed on the per protocol cohort (n=16)
