# Supplementary material for: Safety and High Level Efficacy of the Combination Malaria Vaccine Regimen of RTS,S/AS01B With Chimpanzee Adenovirus 63 and Modified Vaccinia Ankara Vectored Vaccines Expressing ME-TRAP
Source: J Infect Dis. 2016 Jun 15;214(5):772–81. doi: 10.1093/infdis/jiw244 (PMC4978377; doi:10.1093/infdis/jiw244)
Supplement: Supplementary Data [file supp_jiw244_jiw244supp_table11.docx]

| **Instance** | **Group** | **AE** | **Grade** | **Timepoints** | | | | | |
| --- | --- | --- | --- | --- | --- | --- | --- | --- | --- |
| **1** | 1 | Anaemia | 1 | 63 | 70 |  |  |  |  |
| **2** | 2 | Anaemia | 1 | 7 | 63 | 70 | 83 |  |  |
| **3** | 2 | Anaemia | 1 | 7 |  |  |  |  |  |
| **1** | 1 | Leukopenia | 1 | 21 | 35 | 56 | 77 |  |  |
| **1** | 1 | Leukocytosis | 1 | 35 | 63 | 70 |  |  |  |
| **1** | 1 | Thrombocytopenia | 1 | 21 |  |  |  |  |  |
| **1** | 1 | Neutropenia | 1 | 77 |  |  |  |  |  |
| **2** | 1 | Neutropenia | 1 | 7 | 21 | 28 | 35 | 56 | 83 |
| **3** | 1 | Neutropenia | 2 | 77 |  |  |  |  |  |
| **1** | 1 | Lymphopenia | 1 | 21 |  |  |  |  |  |
| **1** | 1 | Eosinophilia | 1 | 35 | 56 | 63 | 70 |  |  |
| **2** | 2 | Eosinophilia | 1 | 35 |  |  |  |  |  |
| **3** | 1 | Eosinophilia | 1 | 7 | 21 | 35 |  |  |  |
| **1** | 1 | Hyponatraemia | 1 | 77 | 83 |  |  |  |  |
| **2** | 1 | Hyponatraemia | 1 | 77 |  |  |  |  |  |
| **1** | 2 | Hypokalaemia | 1 | 56 | 70 |  |  |  |  |
| **2** | 1 | Hypokalaemia | 1 | 7 | 56 |  |  |  |  |
| **3** | 1 | Hypokalaemia | 1 | 70 |  |  |  |  |  |
| **1** | 1 | Uraemia | 1 | 70 |  |  |  |  |  |
| **2** | 2 | Uraemia | 1 | 56 |  |  |  |  |  |
| **3** | 2 | Uraemia | 1 | 63 | 70 |  |  |  |  |
| **1** | 1 | Raised creatinine | 1 | 21 |  |  |  |  |  |
| **1** | 1 | Hyperbilirubinaemia | 1 | 21 | 35 | 63 | 70 | 77 | 83 |
| **2** | 2 | Hyperbilirubinaemia | 1 | 63 |  |  |  |  |  |
| **3** | 1 | Hyperbilirubinaemia | 1 | 83 |  |  |  |  |  |
| **4** | 2 | Hyperbilirubinaemia | 1 | 56 |  |  |  |  |  |
| **5** | 1 | Hyperbilirubinaemia | 1 | 7 | 70 |  |  |  |  |
| **6** | 1 | Hyperbilirubinaemia | 1 | 7 | 35 |  |  |  |  |
| **7** | 2 | Hyperbilirubinaemia | 1 | 63 |  |  |  |  |  |
| **8** | 2 | Hyperbilirubinaemia | 1 | 63 | 70 |  |  |  |  |
| **9** | 1 | Hyperbilirubinaemia | 1 | 7 | 56 | 83 |  |  |  |
| **10** | 1 | Hyperbilirubinaemia | 1 | 83 |  |  |  |  |  |
| **1** | 1 | Raised ALT | 1 | 56 | 63 | 65 | 83 |  |  |
| **1** | 2 | Hypoalbuminaemia | 1 | 35 |  |  |  |  |  |
| **2** | 1 | Hypoalbuminaemia | 3 | 56 |  |  |  |  |  |

Table S11: All abnormal laboratory results within the adverse event range in the pre-CHMI period by severity and timepoint. Each line represents all instances at that severity in a single subject. See supplementary tables in appendix for severity grading criteria by study site. (Grade1 = mild; grade 2 = moderate; grade 3 = severe).
