# Supplementary material for: Safety and High Level Efficacy of the Combination Malaria Vaccine Regimen of RTS,S/AS01B With Chimpanzee Adenovirus 63 and Modified Vaccinia Ankara Vectored Vaccines Expressing ME-TRAP
Source: J Infect Dis. 2016 Jun 15;214(5):772–81. doi: 10.1093/infdis/jiw244 (PMC4978377; doi:10.1093/infdis/jiw244)
Supplement: Supplementary Data [file supp_jiw244_jiw244supp_table12.docx]

| **Instance** | **Group** | **AE** | **Grade** | **Timepoints** | | | | | | | |
| --- | --- | --- | --- | --- | --- | --- | --- | --- | --- | --- | --- |
| **1** | 2 | Anaemia | 1 | C+35 |  |  |  |  |  |  |  |
| **2** | 1 | Anaemia | 1 | C+9 | C+21 | C+35 | C+90 | R+9 | Rdiagnosis | RC+35 | RC+90 |
| **3** | 1 | Anaemia | 1 | C+9 |  |  |  |  |  |  |  |
| **4** | 1 | Anaemia | 1 | C+9 | Diagnosis | C+35 | RC-1 | RC+35 | RC+9 | Rdiagnosis |  |
| **5** | 1 | Anaemia | 1 | C+90 |  |  |  |  |  |  |  |
| **6** | 2 | Anaemia | 1 | C+9 | C+35 | C+90 |  |  |  |  |  |
| **7** | 1 | Anaemia | 1 | Diagnosis | Rdiagnosis | RC+35 |  |  |  |  |  |
| **8** | 1 | Anaemia | 1 | C+9 |  |  |  |  |  |  |  |
| **1** | 2 | Leukopenia | 1 | RC+13 |  |  |  |  |  |  |  |
| **2** | 3 | Leukopenia | 1 | c+9 |  |  |  |  |  |  |  |
| **3** | 1 | Leukopenia | 1 | Rdiagnosis |  |  |  |  |  |  |  |
| **4** | 1 | Leukopenia | 1 | RDiagnosis |  |  |  |  |  |  |  |
| **1** | 2 | Leukocytosis | 1 | C+90 | RC+9 |  |  |  |  |  |  |
| **1** | 3 | Thrombocytopenia | 1 | Diagnosis |  |  |  |  |  |  |  |
| **2** | 2 | Thrombocytopenia | 2 | RC+13 |  |  |  |  |  |  |  |
| **1** | 1 | Neutropenia | 1 | C+9 | Diagnosis | RC-1 | RC+9 | Rdiagnosis |  |  |  |
| **2** | 3 | Neutropenia | 2 | C+9 |  |  |  |  |  |  |  |
| **3** | 4 | Neutropenia | 1 | RC+35 |  |  |  |  |  |  |  |
| **4** | 2 | Neutropenia | 2 | RC+13 |  |  |  |  |  |  |  |
| **1** | 3 | Lymphopenia | 1 | Diagnosis |  |  |  |  |  |  |  |
| **2** | 2 | Lymphopenia | 1 | C+90 |  |  |  |  |  |  |  |
| **3** | 3 | Lymphopenia | 1 | C+9 | C+9 |  |  |  |  |  |  |
| **4** | 1 | Lymphopenia | 1 | Diagnosis |  |  |  |  |  |  |  |
| **5** | 1 | Lymphopenia | 2 | Diagnosis |  |  |  |  |  |  |  |
| **6** | 3 | Lymphopenia | 2 | Diagnosis |  |  |  |  |  |  |  |
| **7** | 1 | Lymphopenia | 2 | EP+1 |  |  |  |  |  |  |  |
| **8** | 2 | Lymphopenia | 2 | Diagnosis |  |  |  |  |  |  |  |
| **9** | 3 | Lymphopenia | 3 | Diagnosis |  |  |  |  |  |  |  |
| **10** | 2 | Lymphopenia | 3 | C+14 |  |  |  |  |  |  |  |
| **11** | 2 | Lymphopenia | 1 | RDiagnosis |  |  |  |  |  |  |  |
| **12** | 4 | Lymphopenia | 1 | RDiagnosis |  |  |  |  |  |  |  |
| **13** | 4 | Lymphopenia | 1 | RDiagnosis |  |  |  |  |  |  |  |
| **14** | 4 | Lymphopenia | 3 | RDiagnosis |  |  |  |  |  |  |  |
| **15** | 2 | Lymphopenia | 3 | RC-1 |  |  |  |  |  |  |  |
| **1** | 1 | Eosinophilia | 1 | C+35 |  |  |  |  |  |  |  |
| **2** | 4 | Eosinophilia | 1 | RC+35 |  |  |  |  |  |  |  |
| **3** | 1 | Eosinophilia | 1 | RC-1 | RC+6 | RC+9 |  |  |  |  |  |
| **4** | 4 | Eosinophilia | 2 | RC+9 |  |  |  |  |  |  |  |
| **1** | 4 | Hypernatraemia | 1 | RC-1 | RC+9 |  |  |  |  |  |  |
| **2** | 4 | Hypernatraemia | 1 | RC+90 |  |  |  |  |  |  |  |
| **3** | 2 | Hypernatraemia | 1 | RC-1 |  |  |  |  |  |  |  |
| **4** | 2 | Hyponatraemia | 1 | RC-1 |  |  |  |  |  |  |  |
| **1** | 4 | Hypokalaemia | 1 | RC+35 |  |  |  |  |  |  |  |
| **2** | 4 | Hypokalaemia | 1 | RC+35 |  |  |  |  |  |  |  |
| **3** | 2 | Hypokalaemia | 1 | Diagnosis |  |  |  |  |  |  |  |
| **4** | 2 | Hypokalaemia | 1 | Diagnosis |  |  |  |  |  |  |  |
| **5** | 3 | Hypokalaemia | 2 | C+90 |  |  |  |  |  |  |  |
| **6** | 2 | Hypokalaemia | 2 | Diagnosis |  |  |  |  |  |  |  |
| **7** | 3 | Hypokalaemia | 2 | C+90 |  |  |  |  |  |  |  |
| **1** | 1 | Uraemia | 1 | C+35 | C+90 | RC+35 |  |  |  |  |  |
| **2** | 1 | Uraemia | 1 | C+9 |  |  |  |  |  |  |  |
| **3** | 2 | Uraemia | 1 | RC+35 |  |  |  |  |  |  |  |
| **1** | 1 | Hyperbilirubinaemia | 1 | C+90 |  |  |  |  |  |  |  |
| **2** | 1 | Hyperbilirubinaemia | 1 | C+9 |  |  |  |  |  |  |  |
| **3** | 3 | Hyperbilirubinaemia | 1 | C+35 |  |  |  |  |  |  |  |
| **4** | 1 | Hyperbilirubinaemia | 2 | C+35 | C+90 |  |  |  |  |  |  |
| **5** | 1 | Hyperbilirubinaemia | 2 | Diagnosis |  |  |  |  |  |  |  |
| **6** | 1 | Hyperbilirubinaemia | 3 | C+9 | C+21 |  |  |  |  |  |  |
| **7** | 2 | Hyperbilirubinaemia | 1 | Re-screen |  |  |  |  |  |  |  |
| **8** | 2 | Hyperbilirubinaemia | 1 | RC-1 | RC+35 | RC+90 |  |  |  |  |  |
| **9** | 2 | Hyperbilirubinaemia | 1 | RC+35 |  |  |  |  |  |  |  |
| **1** | 1 | Raised ALT | 1 | C+9 |  |  |  |  |  |  |  |
| **2** | 2 | Raised ALT | 2 | C+35 |  |  |  |  |  |  |  |
| **3** | 3 | Raised ALT | 2 | C+18 |  |  |  |  |  |  |  |
| **4** | 1 | Raised ALT | 3 | C+90 | C+93 |  |  |  |  |  |  |
| **1** | 1 | Raised Alk Phos | 1 | C+18 |  |  |  |  |  |  |  |

Table S12: All abnormal laboratory results within the adverse event range in the post-CHMI period by severity and timepoint. Each line represents all instances at that severity in a single subject. See supplementary tables in appendix for severity grading criteria by study site. (Grade1 = mild; grade 2 = moderate; grade 3 = severe).
