# Supplementary material for: Safety and High Level Efficacy of the Combination Malaria Vaccine Regimen of RTS,S/AS01B With Chimpanzee Adenovirus 63 and Modified Vaccinia Ankara Vectored Vaccines Expressing ME-TRAP
Source: J Infect Dis. 2016 Jun 15;214(5):772–81. doi: 10.1093/infdis/jiw244 (PMC4978377; doi:10.1093/infdis/jiw244)
Supplement: Supplementary Data [file supp_jiw244_jiw244supp_table13.docx]

|  | **Mean parasite density (parasites per ml) at cycle peaks in all subjects (±SD)** | | | **Mean AUC in infected volunteers across first 3 replication cycles (±SD)** | **Mean fold rise in parasitaemia between cycle peaks in infected volunteers** | |
| --- | --- | --- | --- | --- | --- | --- |
|  | **Cycle 1 (C+7.5)** | **Cycle 2 (9.5)** | **Cycle 3 (11.5)** |  | **Cycle 1 to Cycle 2** | **Cycle 1 to Cycle 3** |
| Group 1 | 0 | 2.7 (±8.7) | 54.5 (±198.8) | 1534.5 (±1651.8) | 16.0 (±15.5) | 306.7 (±383.9) |
| Group 2 | 2.3 (±6.7) | 4.4 (±9.9) | 90.0 (±325.6) | 919.3 (+/-931.1) | 4.4 (±3.9) | 35.6 (±50.5) |
| Group 3 | 87.5 (±97.2) | 410.9 (±279.4) | 2641.3 (±1344.5) | 7906.9 (±3810.1) | 8.7 (±8.7) | 78.7 (±72.1) |

Table S13: Measures of parasite density across the first 3 replication cycles after CHMI (C+6.5 to C+12). Two subjects in Group 3 and one subject in Group 2 were diagnosed and treated for malaria prior to the C+12 visit and therefore do not have a PCR parasitaemia reading for this visit. In these subjects AUC in the third cycle was adjusted according to the proportion of the third cycle duration that they had completed.
