# Supplementary material for: Safety and High Level Efficacy of the Combination Malaria Vaccine Regimen of RTS,S/AS01B With Chimpanzee Adenovirus 63 and Modified Vaccinia Ankara Vectored Vaccines Expressing ME-TRAP
Source: J Infect Dis. 2016 Jun 15;214(5):772–81. doi: 10.1093/infdis/jiw244 (PMC4978377; doi:10.1093/infdis/jiw244)
Supplement: Supplementary Data [file supp_jiw244_jiw244supp_table14.docx]

| **Peptide Name** | **Peptide sequence T9/96** | **Peptide sequence 3D7** | **T9/96 Peptide Pool** | **3D7 Peptide Pool** |
| --- | --- | --- | --- | --- |
| TRAP-1 | MNHLGNVKYLVIVFLIFFDL |  | TT1-10 | TD1-10 |
| TRAP-2 | VIVFLIFFDLFLVNGRDVQN |  | TT1-10 | TD1-10 |
| TRAP-3 | FLVNGRDVQNNIVDEIKYSE | **FLVNGRDVQNNIVDEIKYRE** | TT1-10 | TD1-10 |
| TRAP-4 | NIVDEIKYSEEVCNDQVDLY | **NIVDEIKYREEVCNDEVDLY** | TT1-10 | TD1-10 |
| TRAP-5 | EVCNDQVDLYLLMCSGSIR | **EVCNDEVDLYLLMCSGSIR** | TT1-10 | TD1-10 |
| TRAP-6 | LLMCSGSIRRHNWVNHAVP |  | TT1-10 | TD1-10 |
| TRAP-7 | RHNWVNHAVPLAMKLIQQLN |  | TT1-10 | TD1-10 |
| TRAP-8 | LAMKLIQQLNLNDNAIHLYV | **LAMKLIQQLNLNDNAIHLYA** | TT1-10 | TD1-10 |
| TRAP-9 | LNDNAIHLYVNVFSNNAKEI | **LNDNAIHLYASVFSNNAREI** | TT1-10 | TD1-10 |
| TRAP-10 | LNDNAIHLYVNVFSNNAKEI | **SVFSNNAREIIRLHSDASKN** | TT1-10 | TD1-10 |
| TRAP-11 | IRLHSDASKNKEKALIIIRS | **IRLHSDASKNKEKALIIIKS** | TT11-20 | TD11-20 |
| TRAP-12 | KEKALIIIRSLLSTNLPYGR | **KEKALIIIKSLLSTNLPYGK** | TT11-20 | TD11-20 |
| TRAP-13 | LLSTNLPYGRTNLTDALLQV | **LLSTNLPYGKTNLTDALLQV** | TT11-20 | TD11-20 |
| TRAP-14 | TNLTDALLQVRKHLNDRINR |  | TT11-20 | TD11-20 |
| TRAP-15 | RKHLNDRINRENANQLVVIL |  | TT11-20 | TD11-20 |
| TRAP-16 | ENANQLVVILTDGIPDSIQD |  | TT11-20 | TD11-20 |
| TRAP-17 | TDGIPDSIQDSLKESRKLSD |  | TT11-20 | TD11-20 |
| TRAP-18 | SLKESRKLSDRGVKIAVFGI |  | TT11-20 | TD11-20 |
| TRAP-19 | RGVKIAVFGIGQGINVAFNR |  | TT11-20 | TD11-20 |
| TRAP-20 | GQGINVAFNRFLVGCHPSDG |  | TT11-20 | TD11-20 |
| TRAP-21 | FLVGCHPSDGKCNLYADSAW |  | TT21-30 | TD21-30 |
| TRAP-22 | KCNLYADSAWENVKNVIGPF |  | TT21-30 | TD21-30 |
| TRAP-23 | ENVKNVIGPFMKAVCVEVEK |  | TT21-30 | TD21-30 |
| TRAP-24 | MKAVCVEVEKTASCGVWDEW |  | TT21-30 | TD21-30 |
| TRAP-25 | TASCGVWDEWSPCSVTCGKG |  | TT21-30 | TD21-30 |
| TRAP-26 | SPCSVTCGKGTRSRKREILH |  | TT21-30 | TD21-30 |
| TRAP-27 | TRSRKREILHEGCTSEIQEQ | **TRSRKREILHEGCTSELQEQ** | TT21-30 | TD21-30 |
| TRAP-28 | EGCTSEIQEQCEEERCPPKW | **EGCTSELQEQCEEERCLPKR** | TT21-30 | TD21-30 |
| TRAP-29 | CEEERCPPKWEPLDVPDEPE | **CEEERCLPKREPLDVPDEPE** | TT21-30 | TD21-30 |
| TRAP-30 | EPLDVPDEPEDDQPRPRGDN |  | TT21-30 | TD21-30 |
| TRAP-31 | DDQPRPRGDNSSVQKPEENI | **DDQPRPRGDNFAVEKPNENI** | TT31-40 | TD31-40 |
| TRAP-32 | SSVQKPEENIIDNNPQEPSP | **FAVEKPNENIIDNNPQEPSP** | TT31-40 | TD31-40 |
| TRAP-33 | IDNNPQEPSPNPEEGKDENP | **IDNNPQEPSPNPEEGKGENP** | TT31-40 | TD31-40 |
| TRAP-34 | NPEEGKDENPNGFDLDENPE | **NPEEGKGENPNGFDLDENPE** | TT31-40 | TD31-40 |
| TRAP-35 | NGFDLDENPENPPNPDIPEQ | **NGFDLDENPENPPNPPNPPN** | TT31-40 | TD31-40 |
| TRAP-36 | NPPNPDIPEQKPNIPEDSEK | **NPPNPPNPPNPPNPPNPPNP** | TT31-40 | TD31-40 |
| TRAP-37 | *NONE* | **PPNPPNPPNPDIPEQKPNIP** | TT31-40 | TD31-40 |
| TRAP-38 | DIPEQKPNIPEDSEKEVPSD | **DIPEQKPNIPEDSEKEVPSD** | TT31-40 | TD31-40 |
| TRAP-39 | EDSEKEVPSDVPKNPEDDRE |  | TT31-40 | TD31-40 |
| TRAP-40 | VPKNPEDDREENFDIPKKPE |  | TT31-40 | TD31-40 |
| TRAP-41 | ENFDIPKKPENKHDNQNNLP |  | TT41-50 | TD41-50 |
| TRAP-42 | NKHDNQNNLPNDKSDRNIPY | **NKHDNQNNLPNDKSDRYIPY** | TT41-50 | TD41-50 |
| TRAP-43 | NDKSDRNIPYSPLPPKVLDN | **NDKSDRYIPYSPLAPKVLDN** | TT41-50 | TD41-50 |
| TRAP-44 | SPLPPKVLDNERKQSDPQSQ | **SPLAPKVLDNERKQSDPQSQ** | TT41-50 | TD41-50 |
| TRAP-45 | ERKQSDPQSQDNNGNRHVPN |  | TT41-50 | TD41-50 |
| TRAP-46 | DNNGNRHVPNSEDRETRPHG |  | TT41-50 | TD41-50 |
| TRAP-47 | SEDRETRPHGRNNENRSYNR |  | TT41-50 | TD41-50 |
| TRAP-48 | RNNENRSYNRKYNDTPKHPE |  | TT41-50 | TD41-50 |
| TRAP-49 | KYNDTPKHPEREEHEKPDNN |  | TT41-50 | TD41-50 |
| TRAP-50 | REEHEKPDNNKKKGESDNKY |  | TT41-50 | TD41-50 |
| TRAP-51 | KKKGESDNKYKIAGGIAGGL |  | TT51-57 | TT51-57 |
| TRAP-52 | KIAGGIAGGLALLACAGLAY |  | TT51-57 | TT51-57 |
| TRAP-53 | ALLACAGLAYKFVVPGAATP |  | TT51-57 | TT51-57 |
| TRAP-54 | KFVVPGAATPYAGEPAPFDE |  | TT51-57 | TT51-57 |
| TRAP-55 | YAGEPAPFDETLGEEDKDLD |  | TT51-57 | TT51-57 |
| TRAP-56 | TLGEEDKDLDEPEQFRLPEE |  | TT51-57 | TT51-57 |
| TRAP-57 | EPEQFRLPEENEWN |  | TT51-57 | TT51-57 |

Table S14: TRAP peptide pool format Peptide sequences and residue numbers based on those of the P. falciparum clone T9/96 (GenBank no. CAA31440.1).
