# Supplementary material for: Safety and High Level Efficacy of the Combination Malaria Vaccine Regimen of RTS,S/AS01B With Chimpanzee Adenovirus 63 and Modified Vaccinia Ankara Vectored Vaccines Expressing ME-TRAP
Source: J Infect Dis. 2016 Jun 15;214(5):772–81. doi: 10.1093/infdis/jiw244 (PMC4978377; doi:10.1093/infdis/jiw244)
Supplement: Supplementary Data [file supp_jiw244_jiw244supp_table15.docx]

| **Peptide Name** | **Peptide sequence** | **Antigen** |
| --- | --- | --- |
| st8 | MINAYLDKL | STARP |
| ls50 | ISKYEDEI | LSA1 |
| pb9 | SYIPSAEKI | *P. berghei CSP* |
| ls8 | KPNDKSLY | LSA1 |
| cp26 | KPKDELDY | CSP |
| ls6 | KPIVQYDNF | LSA1 |
| tr42/43 | ASKNKEKALII | TRAP |
| tr39 | GIAGGLALL | TRAP |
| cp6 | MNPNDPNRNV | CSP |
| tr26 | HLGNVKYLV | TRAP |
| ls53 | KSLYDEHI | LSA1 |
| tr29 | LLMCSGSI | TRAP |
| csp | DPNANPNVDPNANPNV | CSP |
| 38H BCG | QVHFQPLPPAVVKL | BCG |
| FTTp | QFIKANSKFIGITE | TT |
| cp39 | YLNKIQNSL | CSP |
| la72 | MEKLKELEK | LSA3 |
| ex23 | ATSVLAGL | EXP1 |
| nanp | NANPNANPNANPNANP | CSP |
| trapAM | DEWSPCSVTCGKGTRSRKRE | TRAP |

Table S15: ME peptide pool format
