# Supplementary material for: Safety and High Level Efficacy of the Combination Malaria Vaccine Regimen of RTS,S/AS01B With Chimpanzee Adenovirus 63 and Modified Vaccinia Ankara Vectored Vaccines Expressing ME-TRAP
Source: J Infect Dis. 2016 Jun 15;214(5):772–81. doi: 10.1093/infdis/jiw244 (PMC4978377; doi:10.1093/infdis/jiw244)
Supplement: Supplementary Data [file supp_jiw244_jiw244supp_table16.docx]

| **Peptide number** | **Amino acid sequence** |  |
| --- | --- | --- |
| **NANP and conserved region peptides pool** | | |
| **1** | MMAP DPNANPNANPN |  |
| **2** | NANP NANPNANPNAN |  |
| **3** | DPNA NPNANPNKNNQ |  |
| **4** | NPNA NPNKNNQGNGQ |  |
| **5** | NPNK NNQGNGQGHNM |  |
| **6** | NNQG NGQGHNMPNDP |  |
| **7** | NGQG HNMPNDPNRNV |  |
| **8** | HNMP NDPNRNVDENA |  |
| **9** | NDPN RNVDENANANS |  |
| **10** | RNVD ENANANSAVKN |  |
| **11** | ENAN ANSAVKNNNNE |  |
| **TH2R region peptides pool** | | |
| **12** | ANSA VKNNNNEEPSD |  |
| **13** | VKNN NNEEPSDKHIK |  |
| **14** | NNEE PSDKHIKEYLN |  |
| **15** | PSDK HIKEYLNKIQN |  |
| **16** | HIKE YLNKIQNSLST |  |
| **17** | YLNK IQNSLSTEWSP |  |
| **18** | IQNS LSTEWSPCSVT |  |
| **19** | LSTE WSPCSVTCGNG |  |
| **TH3R/CS.T3T region peptides pool** | | |
| **20** | WSPC SVTCGNGIQVR |  |
| **21** | SVTC GNGIQVRIKPG |  |
| **22** | GNGI QVRIKPGSANK |  |
| **23** | QVRI KPGSANKPKDE |  |
| **24** | KPGS ANKPKDELDYA |  |
| **25** | ANKP KDELDYANDIE |  |
| **26** | KDEL DYANDIEKKIC |  |
| **27** | DYAN DIEKKICKMEK |  |
| **28** | DIEK KICKMEKCSSV |  |
| **29** | KICK MEKCSSVFNVV |  |
| **30** | MEKC SSVFNVVNSSI |  |
| **31** | KCSS VFNVVNSSIGL |  |

Table S16: CS peptide pool format [11]

| **Pool** | **CSP aa** | **No. peptides** |
| --- | --- | --- |
| **Cp1** | **1-39** | **7** |
| **Cp2** | **29-71** | **8** |
| **Cp3** | **61-107** | **9** |

*Peptide sequences and residue numbers were based on those of the P. falciparum clone 3D7 (GenBank no. X15363). Series of 15 amino acid peptide sequences overlapping by 11 amino acids.*
