# Supplementary material for: Safety and High Level Efficacy of the Combination Malaria Vaccine Regimen of RTS,S/AS01B With Chimpanzee Adenovirus 63 and Modified Vaccinia Ankara Vectored Vaccines Expressing ME-TRAP
Source: J Infect Dis. 2016 Jun 15;214(5):772–81. doi: 10.1093/infdis/jiw244 (PMC4978377; doi:10.1093/infdis/jiw244)
Supplement: Supplementary Data [file supp_jiw244_jiw244supp_table17.docx]

| Antibody | Fluorochrome  /Dye | Clone | Supplier | Product code | Final Dilution |
| --- | --- | --- | --- | --- | --- |
|  | LIVE/DEAD  (Amine reactive dye) | AQUA | Life Technologies | L34955 | 1:400 |
| CD3 | Alexa Fluor 700 | OKT3 | eBioscience | 56-0037-42 | 1:100 |
| CD4 | Allophycocyanin (APC) | RPA-T4 | eBioscience | 17-0049-73 | 1:50 |
| CD8 | APC-eFluor780 | RPA-T8 | eBioscience | 47-0088-42 | 1:25 |
| CD14 | eFluor 450 | 61D3 | eBioscience | 48-0149 | 1:100 |
| CD19 | eFluor 450 | HIB19 | eBioscience | 48-0199 | 1:100 |
| CD107a | PE-Cy5 | eBioH4A3 | eBioscience | 15-1079-42 | 1:100 |
| IFNγ | FITC | 4S.B3 | eBioscience | 11-7319-82 | 1:200 |
| IL-2 | PE | MQ1-17H12 | eBioscience | 12-7029-82 | 1:100 |
| TNFα | PE-Cy7 | MAb11 | eBioscience | 25-7349-82 | 1:1000 |

Table S17: Antibodies used for flow cytometry. CD107a was added at the start of the 18-hour stimulation, LIVE/DEAD was surface-stained. All other markers were stained after permeabilisation.
